# Ovarian Preservation Before Puberty: Surgical Management of Adnexal Torsion in Prepubertal and Prepubertal-Predominant Paediatric Cohorts—A Systematic Review and Meta-Analysis

**DOI:** 10.3390/jcm15218678

**Published:** 2026-11-06

**Authors:** Hanadi Bakhsh

**Affiliations:** 1 Obstetrics and Gynecology Department, College of Medicine, Princess Nourah bint Abdulrahman University, Riyadh 11564, Saudi Arabia; habakhsh@pnu.edu.sa; 2 Department of Obstetrics and Gynecology, King Abdullah bin Abdulaziz University Hospital, Princess Nourah bint Abdulrahman University, Riyadh 13412, Saudi Arabia

**Keywords:** adnexal torsion, ovarian torsion, prepubertal, premenarchal, ovarian-sparing surgery, oophorectomy, detorsion, fertility preservation, paediatric and adolescent gynaecology, meta-analysis

## Abstract

**Background/Objectives**: Adnexal torsion is a gynaecological emergency in which ovarian preservation or removal is decided intraoperatively. Prepubertal girls have the longest reproductive and endocrine horizon, yet they are commonly pooled with postmenarchal adolescents. This review estimated how often torsion episodes in prepubertal and prepubertal-predominant paediatric cohorts are managed by oophorectomy, together with imaging findings after ovarian-sparing surgery, recurrence and diagnosed malignancy. **Methods**: A PRISMA 2020 systematic review and meta-analysis (PROSPERO CRD420261496245) searched Europe PMC (including MEDLINE), OpenAlex, preprint servers, ClinicalTrials.gov and citation networks to 2 September 2026, with an update search to 12 October 2026; Embase, Scopus, Web of Science, CINAHL and CENTRAL were not searched. Eligible cohorts documented premenarchal status (direct evidence) or had a central age of 12.0 years or younger (indirect evidence). Proportions were pooled with a binomial–normal generalised linear mixed model; JBI appraisal and GRADE were applied. **Results**: Thirty studies were included. Across 14 cohorts (1011 episodes), 42.8% (95% CI 28.1–58.9) were managed by oophorectomy, with extreme heterogeneity (I^2^ = 94.0%; 95% prediction interval 6.0–89.9%); the estimate was 33.4% (8.5–69.8) in the 4 cohorts with documented premenarchal status and 24.8% (14.4–37.3) in the 7 cohorts meeting all appraisal items. Follicular activity or normal morphology was seen on follow-up imaging in 89.9% (81.9–95.9) of 234 imaged adnexa after ovarian-sparing surgery. Recurrence was 12.9% (6.6–21.0; 11 cohorts) and diagnosed malignancy 2.7% (1.0–4.6; 7 cohorts). Certainty was very low for ovarian loss and imaging findings and low for recurrence and malignancy. **Conclusions**: Reported oophorectomy rates in prepubertal-predominant cohorts vary widely and often exceed the low prevalence of diagnosed malignancy, but these data cannot establish how many removals were avoidable. The findings support alignment with existing ovarian-conservation guidance and better reporting of menarchal status, indications for removal and functional follow-up.

## 1. Introduction

Adnexal torsion—rotation of the ovary, fallopian tube or both around the vascular pedicle—is among the few true emergencies in paediatric and adolescent gynaecology. Nationally representative United States data place the incidence at approximately 4.9 per 100,000 females aged 1 to 20 years, making it an uncommon but far from rare cause of the acute paediatric abdomen [1]. Its clinical importance is disproportionate to its frequency, because the operative decision is binary, is taken within minutes of entering the abdomen, and cannot be reversed: the surgeon either untwists the adnexa and leaves it in situ or removes it.

The prepubertal ovary is not simply a smaller version of the adult organ, and prepubertal torsion is not simply paediatric torsion at a younger age. In girls who have not yet reached menarche, torsion arises disproportionately on a morphologically normal adnexa, in the absence of any cyst or neoplasm, and is attributed to a long and lax utero-ovarian ligament, a mobile mesosalpinx and the relatively high position of the ovary in the shallow prepubertal pelvis [2,3]. Because no mass is present to explain the pain, and because the symptoms—intermittent abdominal pain, nausea, vomiting and occasionally urinary complaints—are shared with appendicitis, gastroenteritis and constipation, the diagnosis is delayed more often and for longer than in older girls [4,5]. The same anatomical predisposition that produced the first episode persists after operation, so the premenarchal ovary that has torted once is at substantially elevated risk of doing so again [6].

Historically, the torted adnexa were removed. The rationale rested on three propositions: that a black or blue ovary is necrotic and cannot recover; that detorsion of an infarcted organ risks thromboembolism, peritonitis or sepsis; and that an enlarged ovary may harbour a malignancy that conservative surgery would disseminate [7,8]. Each has since been challenged. A systematic review commissioned by the American Pediatric Surgical Association concluded in 2018 that detorsion with ovarian preservation should be standard care in children and adolescents [9], and in 2019, the American College of Obstetricians and Gynecologists issued Committee Opinion No. 783, which states that conservative management is appropriate irrespective of the intraoperative appearance of the ovary and that oophorectomy should be reserved for cases in which the ovary is clearly non-viable or malignancy is strongly suspected [10].

The clinical stakes of getting this decision wrong are highest before puberty. Unilateral oophorectomy removes approximately half of the primordial follicle pool before any of it has been used. This is not equivalent to halving future fertility: follicle number, circulating markers of ovarian reserve such as anti-Müllerian hormone, reproductive lifespan and the probability of live birth are distinct outcomes. Population-based data indicate that the likelihood of eventual childbirth with one ovary remains substantial, although unilateral oophorectomy has been associated with reduced fertility and earlier ovarian ageing [11,12]. Compensatory hypertrophy of the remaining ovary is well documented but incomplete [13]. More importantly, a girl who loses one ovary before menarche depends on a single gonad for the whole of her subsequent reproductive and endocrine life, while remaining exposed to the same anatomical risk of contralateral torsion that produced the first event.

Despite two decades of consistent recommendations, most syntheses of the literature pool prepubertal girls with postmenarchal adolescents and young adults, in whom the prevalence of underlying cysts is higher, the diagnosis is easier, the recurrence risk is lower and the reproductive horizon is shorter [9,14,15]. As a consequence, the quantity that matters most to the prepubertal patient—how often her ovary is actually removed, and how that compares with what could be preserved and with what genuinely needs to be removed—has not been estimated, and the evidence that directly documents menarchal status is sparse. This review was designed to answer four questions:

**RQ1.** What proportion of surgically confirmed torsion episodes in prepubertal girls, and in paediatric cohorts presumed to be predominantly prepubertal, are managed by oophorectomy, and does this vary by era, geographical region or the way the cohort defines prepubertal status?

**RQ2.** Among adnexa managed by ovarian-sparing surgery, what proportion demonstrate follicular activity or normal morphology on follow-up imaging, and what is known about functional recovery?

**RQ3.** What is the risk of torsion recurrence after ovarian-sparing surgery in this population, and what does the available evidence show about oophoropexy?

**RQ4.** What is the prevalence of diagnosed ovarian malignancy in prepubertal torsion, and how was it ascertained?

Each question is answered in a correspondingly numbered subsection of the Results and revisited in the Principal Findings section of the Discussion.

## 2. Materials and Methods

### 2.1. Protocol, Registration and Reporting

This review was conducted according to a protocol registered with the International Prospective Register of Systematic Reviews (PROSPERO: CRD420261496245) and is reported in accordance with the Preferred Reporting Items for Systematic Reviews and Meta-Analyses (PRISMA) 2020 statement [16]; the completed checklist is provided as Supplementary File S4. The registered protocol is publicly accessible through the PROSPERO record. Amendments made after registration, all in response to peer review, are described below and cross-referenced in Supplementary File S4 (item 24c): re-framing of the population into direct and indirect evidence with additional age-threshold analyses; withdrawal of the derived ‘preventable oophorectomy’ metrics; domain-based quality sensitivity analyses; meta-regression on the treatment-period midpoint; recalculation of prediction intervals; and an update search. Because the review used only aggregate data from published studies, institutional review board approval and informed consent were not required.

### 2.2. Eligibility Criteria

The question was framed using the CoCoPop structure. The *Condition* was intraoperatively confirmed adnexal torsion, comprising isolated ovarian torsion and combined tubo-ovarian torsion. The *Context* was emergency or urgent operative management, by laparoscopy or laparotomy, in any hospital setting and any country. The *Population* was girls who had not attained menarche. Prepubertal (no secondary sexual development) and premenarchal (menarche not yet reached) are not synonymous, because pubertal development usually begins some years before menarche. Included studies recorded menarche rather than pubertal stage, so eligibility was operationalised on premenarchal status, and ‘prepubertal’ is used in this review as a descriptor of the target population. Because menarchal status is inconsistently documented, three operational routes to eligibility were pre-specified and, at revision, grouped into two evidence tiers. Direct evidence comprised (i) studies enrolling exclusively premenarchal girls and (ii) studies reporting a separately extractable premenarchal subgroup. Indirect evidence comprised (iii) paediatric cohorts whose reported central age (mean or median) was 12.0 years or younger, described as prepubertal-predominant. The threshold was chosen because the median age at menarche in United States girls is about 12.4 years, a cohort with a central age at or below this value will nonetheless include some postmenarchal girls. Indirect-evidence cohorts were, therefore, analysed separately, tested at stricter thresholds and rated down for indirectness.

Eligible designs were cohort studies and consecutive case series enrolling at least 10 torsion episodes and reporting at least one review outcome; the minimum of 10 episodes was used to exclude case reports and very small series, in which proportions are unstable and selectively reported. Reports were excluded if they were case reports or series of fewer than 10 episodes; narrative or systematic reviews, guidelines, editorials or conference abstracts; studies of isolated fallopian tube torsion without ovarian involvement; studies confined to postmenarchal, adult or pregnant populations; neonatal ovarian cyst series without confirmed torsion surgery data; or diagnostic-accuracy and prediction-model studies that did not report surgical management. Cohorts whose population was selected on the procedure received—for example, series composed exclusively of patients treated by detorsion—were ineligible for the primary analysis of the oophorectomy proportion, because such designs cannot estimate that proportion, but remained eligible for the analyses of imaging findings after ovarian-sparing surgery and recurrence. Where two reports described overlapping patients from the same institution and period, the report providing the more complete data for the outcome in question was retained and the other excluded from that analysis. No language or date restrictions were applied.

**Outcomes.** The primary outcome was the proportion of torsion episodes managed by oophorectomy, salpingo-oophorectomy or adnexectomy (collectively, ovarian loss). Secondary outcomes were the proportion of adnexa managed by ovarian-sparing surgery and imaged at follow-up that demonstrated follicular activity or normal ovarian morphology on ultrasound (a composite sonographic outcome; its components—follicles, size or morphology, Doppler flow and atrophy—were also tabulated separately); the proportion of girls experiencing at least one recurrent torsion episode; and the proportion of torsion episodes with a diagnosed ovarian malignancy, that is, malignancy confirmed histologically in any resected or biopsied tissue. Ovaries preserved without excision or biopsy had no histological verification, so this outcome measures diagnosed rather than histologically excluded malignancy. Oophorectomy, salpingo-oophorectomy and adnexectomy were combined solely because each entails loss of the ovary. The unit of analysis was the torsion episode or affected adnexa, as reported by each study: bilateral torsion contributed one episode per affected adnexa and recurrent episodes contributed separately. Because individual-level data were unavailable, within-patient correlation could not be modelled; in the two primary cohorts reporting both units, episodes exceeded patients modestly (45 episodes in 40 girls [17]; 39 in 32 [18]).

### 2.3. Information Sources and Search Strategy

The search combined three concept blocks—condition, population and surgical management—each expanded with controlled vocabulary, free-text synonyms, spelling variants and truncation. The master strategy was as follows:

*(“ovarian torsion” OR “adnexal torsion” OR “tubo-ovarian torsion” OR “torsion of the ovary”) AND (prepubertal OR pre-pubertal OR prepubescent OR premenarchal OR premenarcheal OR child OR children OR childhood OR paediatric OR pediatric OR girl OR girls OR infant OR infants OR neonatal OR neonate) AND (surgery OR surgical OR laparoscopy OR laparoscopic OR laparotomy OR detorsion OR oophorectomy OR ovariectomy OR salpingo-oophorectomy OR adnexectomy OR oophoropexy OR cystectomy OR “ovarian preservation” OR “ovarian sparing” OR “ovary sparing” OR conservative OR salvage).*

The strategy was executed on 2 September 2026 against Europe PMC, which indexes MEDLINE/PubMed (1985 records), PubMed Central content not indexed in MEDLINE (77 records) and preprint servers (13 records); OpenAlex, a multidisciplinary bibliographic index used to capture records outside MEDLINE (737 records, of which 334 carried no PubMed identifier); and the ClinicalTrials.gov registry (5 records). Crossref was used to verify the bibliographic metadata and digital object identifier of every citation. The search was supplemented by backward citation chasing of all included reports and of previous syntheses of paediatric ovarian torsion, and by forward citation chasing of the most frequently cited included cohorts. Embase, CINAHL, Scopus, Web of Science and Cochrane CENTRAL were not searched, because institutional access to these databases was not available; the translations of the strategy for these databases in Supplementary File S1 are provided for reproducibility only and do not represent searches that were run. This review is, therefore, a restricted-source review. Grey literature was sought through preprint servers, ClinicalTrials.gov and citation chasing; dissertations and conference proceedings were not actively searched, and no language limits were applied. Requiring a surgical-management concept may have reduced sensitivity for reports that describe operative outcomes without these terms in searchable fields. An update search of Europe PMC was run on 12 October 2026 for records first published from 1 September 2026, using the condition terms alone (without the population or surgical blocks); it retrieved 21 records, none of which was eligible.

### 2.4. Study Selection

Records were exported and deduplicated by a scripted comparison of digital object identifier, PubMed identifier and normalised title; 403 duplicate records were removed. Screening proceeded in two stages—title and abstract, then full text—against the eligibility criteria. All screening, full-text assessment, data extraction and critical appraisal were performed by a single reviewer (H.B.); there was no independent duplicate screening, extraction or appraisal. Single-reviewer procedures are known to miss eligible studies and to increase extraction errors [19,20], and this is acknowledged as a limitation. To reduce error, every study advanced to full-text assessment was checked against the primary source record, and every extracted datum was traced to the sentence in the source report from which it was taken. During revision, every count contributing to a pooled analysis was re-verified against the published abstract or, where accessible, the full text; this identified and corrected two extraction errors in the imaging outcome (Mandarano et al. [21], 8 of 13 rather than 11 of 18 adnexa; Oskaylı et al. [22], 20 rather than 17 of 22 under the composite definition). Where reports from the same institution overlapped in time, each synthesis used only one of them; overlap between multicentre and single-centre Italian series could not be excluded. Potentially overlapping reports were evaluated by outcome, and one report was retained for each overlapping cohort and outcome. The available screening and eligibility log, including recorded full-text decisions and exclusion reasons, is provided as Supplementary File S3.

### 2.5. Data Extraction

A structured extraction sheet captured first author, year, country, study period, design, single- or multicentre status, number of patients and of torsion episodes, age (central value and dispersion), route to prepubertal eligibility, surgical approach, number of episodes managed by oophorectomy and by ovarian-sparing surgery, oophoropexy use, follow-up imaging findings, recurrence, histology including malignancy, and complications. Where a study reported both patients and episodes, the episode was used as the denominator, and this is stated in Table 1. Where the number of events was reported only as a percentage, the count was reconstructed and cross-checked against every other figure in the report. For follow-up imaging, the number of adnexa conserved, the number imaged, the modality, the timing and the definition of a favourable result were extracted (Section 3.4). For malignancy, per-study diagnosed malignancy counts are supplied in Supplementary File S2d; limitations of histological ascertainment and the handling of conserved ovaries are described in Section 3.6. Available outcome-specific extraction sheets are provided in Supplementary File S2a–S2d.

### 2.6. Methodological Quality

All included studies, whatever their design label (retrospective cohort, before–after or case–control), contributed only single-group proportions to this review; no comparative effect was extracted. Methodological quality was, therefore, appraised with the ten-item Joanna Briggs Institute (JBI) critical appraisal checklist for case series [44], applied to each study as the source of a single-group proportion. The items most relevant to bias in a proportion—consecutive inclusion (item 4), complete inclusion (item 5) and completeness of outcome and follow-up reporting (item 8)—were identified as key domains. Each item was judged yes, unclear or no, and judgements are presented at item level (Supplementary File S2g). The number of items rated yes is shown descriptively in Table 1 but was not used as a summary quality score, because summary scores weight heterogeneous domains equally and can conceal the domains that matter [45]. No study was excluded on the basis of its appraisal. The pre-specified sensitivity analysis restricted to studies rated yes on all ten items is retained for transparency; two domain-based sensitivity analyses, restricted to studies rated yes on both selection items (4 and 5) and to studies rated yes on outcome reporting (item 8), were added post hoc at revision.

### 2.7. Data Synthesis and Statistical Analysis

The primary model for pooling proportions was a binomial–normal generalised linear mixed model with a random study intercept on the logit scale [46], estimated by maximum likelihood with adaptive Gauss–Hermite quadrature (25 nodes) using the metafor (version 4.4-0) and lme4 (version 1.1-35.1) packages in R 4.3.3 [47,48]. Estimates were verified by direct numerical integration of the marginal likelihood. This model uses the exact within-study binomial likelihood, so studies with zero or all events were included without continuity correction. Confidence intervals for the pooled logit were obtained by profile likelihood and back-transformed to the proportion scale.

Two transformation-based random-effects models were pre-specified as sensitivity analyses: an inverse-variance model on the logit scale with between-study variance estimated by restricted maximum likelihood and confidence intervals derived using the Hartung–Knapp–Sidik–Jonkman correction [49], and a Freeman–Tukey double-arcsine model back-transformed at the harmonic mean sample size [50]; in the logit models, 0.5 was added to the cells of studies with zero or all events. A DerSimonian–Laird model without the Hartung–Knapp correction was also fitted for comparability with the older literature [51].

Heterogeneity was quantified by Cochran Q, the I^2^ statistic with the conventional 25%, 50% and 75% thresholds, and the between-study standard deviation τ [52]. Because I^2^ describes the proportion of variability attributable to heterogeneity rather than its magnitude, a 95% prediction interval was computed for every synthesis to express the range of proportions a new comparable cohort might be expected to show [53]. Prediction intervals were calculated as $\hat{µ}\pm t\left(k-2\right)\times \surd \left({\hat{\tau }}^{2}+\mathrm{SE}{\left(\hat{µ}\right)}^{2}\right)$ on the logit scale and back-transformed [54]; where ${\left(\hat{µ}\right)}^{2}$ was estimated as zero, the interval reflects the standard error of the pooled estimate alone.

Three subgroup analyses were pre-specified: route to prepubertal eligibility (direct versus indirect evidence), publication era (2005–2012, 2013–2018, 2019–2026) and geographical region; between-subgroup differences were tested by likelihood-ratio tests in the generalised linear mixed model. At revision, the indirect evidence was additionally restricted to stricter central-age thresholds (11.0, 10.5 and 10.0 years). To approximate the proportion of postmenarchal girls in indirect-evidence cohorts, an illustrative model assumed a normal age distribution around each cohort’s central age (standard deviation 2.5–3.5 years) and a normal distribution of age at menarche (median 12.43 years [55], standard deviation 1.2 years). Mixed-effects meta-regression with restricted maximum likelihood and Knapp–Hartung adjustment examined year of publication as a continuous moderator and, because publication year may poorly reflect when surgery took place, the midpoint of each study’s treatment period in the cohorts that reported it; cohort size and eligibility route were secondary moderators. The comparison between the primary cohorts and the broader paediatric cohorts was tested formally within a single model. Small-study effects were explored by contour-enhanced funnel plot, the Egger regression test on the logit scale, the Peters test and the Duval and Tweedie trim-and-fill procedure [56,57,58]; because these methods have low power and uncertain validity for single proportions with few studies and high heterogeneity [59], they were treated as exploratory and were not used to rule out reporting bias. Robustness was assessed by leave-one-out analysis. A pre-specified sensitivity analysis extended the primary synthesis to all unselected paediatric torsion cohorts, including those whose central age could not be established as 12.0 years or younger.

Analyses were performed in R 4.3.3 using metafor 4.4-0 and lme4 1.1-35.1; figures were drawn in Python 3. The available study-level extraction data, critical appraisal judgements and statistical summary results are provided in Supplementary File S2 (sheets S2a–S2h). All statistical tests were two-sided with α = 0.05.

### 2.8. Exploratory Comparison of Oophorectomy and Diagnosed Malignancy

The ‘preventable oophorectomy fraction’ and ‘number needed to change practice’ specified in the original submission were withdrawn at revision, because they subtracted estimates obtained from different sets of studies and implied a counterfactual, causal effect that observational single-arm data cannot support. Instead, in the cohorts that reported both the operation performed and diagnosed malignancy on the same denominator, the within-cohort difference between the two proportions was summarised descriptively, with uncertainty propagated jointly by a cluster bootstrap (5000 resamples of cohorts with parametric resampling of events within cohorts) and, separately, by a random-effects risk-difference model with Hartung–Knapp–Sidik–Jonkman adjustment. This difference is not an estimate of avoidable oophorectomy: non-malignant indications for removal (for example, an ovary replaced by a mass, autoamputation, uncontrollable haemorrhage or technical impossibility of preservation) were not reported at patient level, and nothing is known about whether removed ovaries would have recovered. Conversely, malignancy does not always require primary oophorectomy, because fertility-sparing surgery is possible for selected malignant or borderline tumours.

### 2.9. Certainty of the Evidence

Certainty was rated for each outcome using GRADE as adapted for estimates of event rates (proportions) from observational evidence [60,61]. Under this approach, bodies of evidence from observational series start at high certainty, because randomisation is not required to estimate how often an event occurs, and are rated down for risk of bias, inconsistency, indirectness, imprecision and publication bias. Inconsistency was judged principally on the width of the 95% prediction interval: rated down one level when it spanned 40–70 percentage points and two levels when it spanned more than 70. Indirectness [62] was judged on the proportion of the evidence derived from cohorts without documented premenarchal status (rated down when this exceeded half of the denominator) and on the use of surrogate endpoints. Imprecision was judged on whether the limits of the confidence interval would support different clinical interpretations; where inconsistency had already been rated down for the same uncertainty, it was not counted again. Publication bias was judged on study-level considerations rather than on statistical tests alone. Every judgement is explained in the footnotes to the GRADE evidence profile (Section 3.10).

## 3. Results

### 3.1. Study Selection

**Figure 1:**
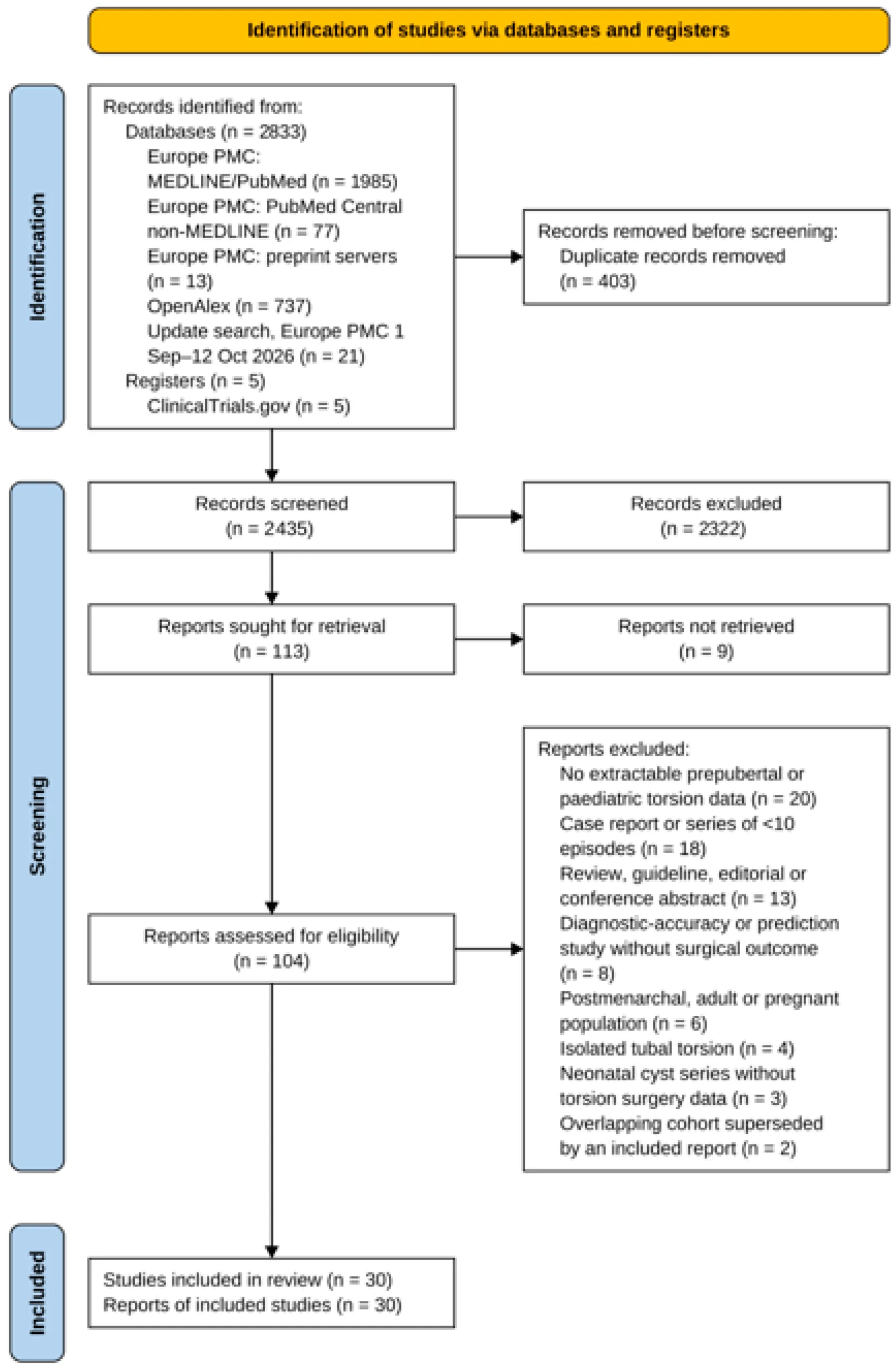
PRISMA 2020 flow diagram of study identification, screening, eligibility assessment and inclusion, including the update search.

The search returned 2817 records, of which 403 were duplicates. After screening 2414 titles and abstracts, 113 reports were sought for retrieval; 9 could not be obtained in full text. Of the 104 reports assessed for eligibility, 74 were excluded, most commonly because no prepubertal, premenarchal or age-stratified paediatric torsion data could be extracted (n = 20) or because the report was a case report or series of fewer than 10 torsion episodes (n = 18). Thirty studies met the eligibility criteria (Figure 1). The update search to 12 October 2026 retrieved 21 further records, none of them eligible. Fourteen contributed to the primary synthesis of the oophorectomy proportion, twelve to imaging findings after ovarian-sparing surgery, eleven to recurrence and seven to malignancy; several studies contributed to more than one synthesis.

### 3.2. Characteristics of the Included Studies

The 30 included studies were published between 2005 and 2026 and were conducted in 12 countries: 11 in North America, 9 in Europe, 8 in the Middle East and East Asia and 2 in South Asia. All were retrospective; 27 were single-centre cohorts or consecutive series and 3 were multicentre collaborations, the largest of which contributed 502 prepubertal girls from 30 French paediatric surgical centres. Study periods ranged from 4 to 26 years (inclusive). The 14 cohorts contributing to the primary analysis comprised 1011 torsion episodes; 4 of these provided direct evidence, enrolling premenarchal girls exclusively or reporting an extractable premenarchal subgroup (195 episodes, 19.3%), and 10 provided indirect evidence as paediatric cohorts with a reported central age of 12.0 years or younger (816 episodes, 80.7%). None of the indirect-evidence cohorts reported the age distribution or menarchal status of its participants. Under the illustrative assumptions described in Section 2.7, approximately 26–31% of girls in the indirect-evidence cohorts, and 21–25% of all 1011 episodes, would have been postmenarchal (these estimates are illustrative, depend on the stated distributional assumptions and are not observed menarchal counts). Study characteristics are summarised in Table 1.

JBI, Joanna Briggs Institute critical appraisal checklist for case series (number of the ten items rated “yes”, shown descriptively; item-level judgements are given in Supplementary File S2g and were not combined into a quality score). NR, not reported. OP, oophoropexy. Eligibility route indicates how the study met the prepubertal population criterion: “Premenarchal cohort” = all participants premenarchal; “Premenarchal subgroup” = separately extractable premenarchal data; “Central age ≤ 12 y” = paediatric cohort with reported mean or median age of 12.0 years or younger; “Paediatric (sensitivity)” = unselected paediatric cohort contributing only to the sensitivity analysis; remaining entries contributed only to the specified secondary outcome. “Premenarchal cohort” and “Premenarchal subgroup” constitute direct evidence; “Central age ≤ 12 y” constitutes indirect evidence. The torsion-episode column gives patients where episodes were not reported (Oltmann, 2010 [7], Smorgick, 2016 [6], Comeau, 2017 [33], Gluska, 2026 [5]); for Defert, 2025 [4] it gives the 482 girls without oophoropexy analysed for recurrence (502 enrolled). Study characteristics are presented in Table 1; the available outcome-specific extraction data are provided in Supplementary File S2a–S2d.

### 3.3. RQ1: Proportion Undergoing Oophorectomy

**Figure 2:**
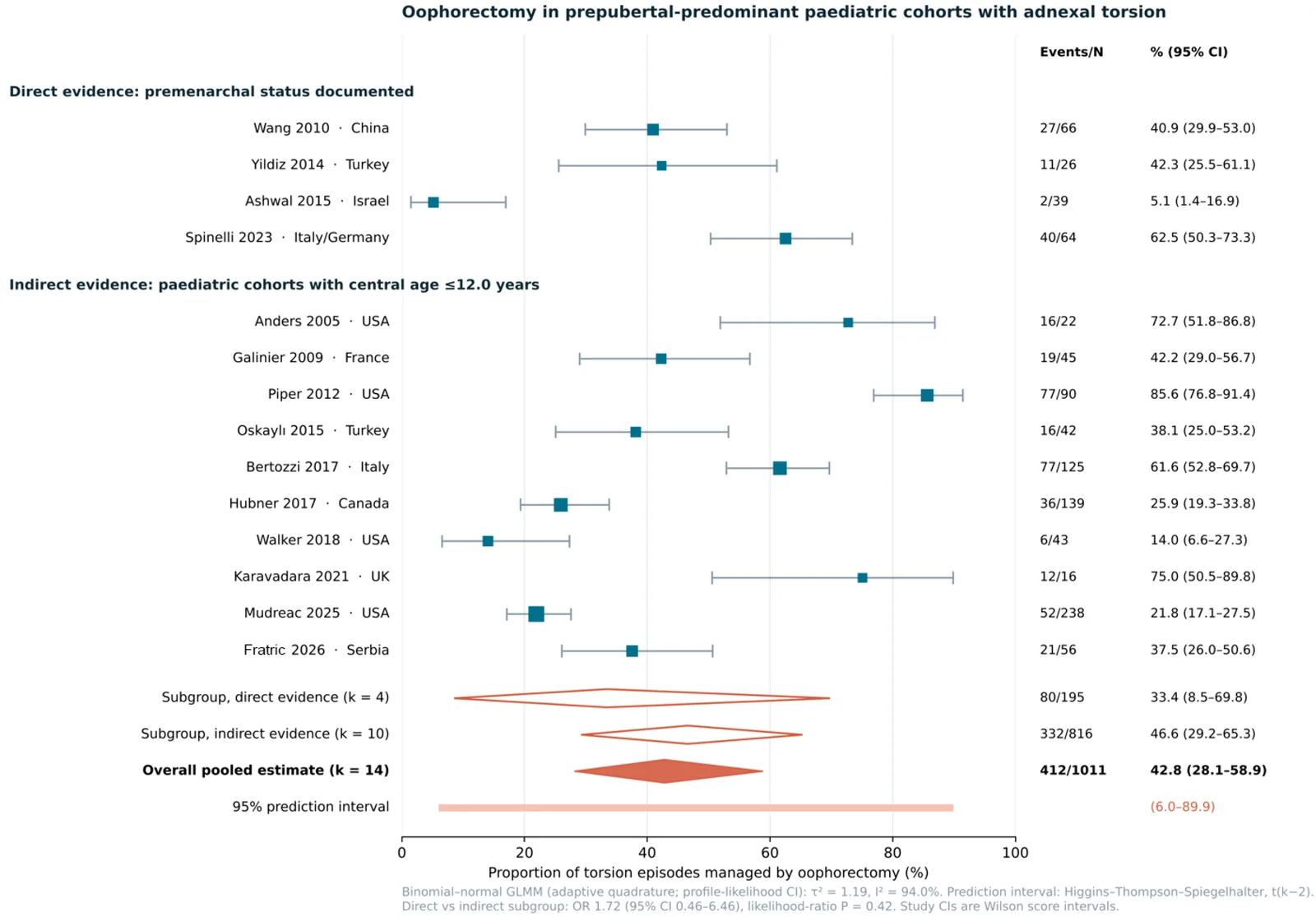
Forest plot of the proportion of torsion episodes managed by oophorectomy in prepubertal and prepubertal-predominant paediatric cohorts, stratified into direct and indirect evidence. Marker area is proportional to cohort size; horizontal lines are Wilson score intervals; open diamonds are subgroup estimates and the filled diamond is the overall pooled estimate from the binomial–normal generalised linear mixed model; the shaded bar is the 95% prediction interval.

Across 14 cohorts and 1011 torsion episodes, 412 (crude 40.8%) were managed by oophorectomy, salpingo-oophorectomy or adnexectomy. The pooled proportion under the binomial–normal generalised linear mixed model was 42.8% (95% CI 28.1–58.9). Between-study heterogeneity was very high (τ^2^ = 1.19; τ = 1.09; I^2^ = 94.0%; Cochran Q = 167.6, df = 13, *p* < 0.001), and the 95% prediction interval spanned 6.0% to 89.9% (Figure 2). The pooled proportion, therefore, describes the centre of a very wide distribution rather than a rate that can be expected in any particular service.

Study-level proportions ranged from 5.1% (2 of 39 episodes; Ashwal et al., Israel [18]) to 85.6% (77 of 90; Piper et al., United States [26]). The alternative models gave similar pooled estimates: 43.3% (27.8–60.2) under the logit random-effects model with the Hartung–Knapp–Sidik–Jonkman correction, 43.4% (30.1–57.3) under the Freeman–Tukey double-arcsine model and 43.4% (31.0–56.6) under an uncorrected DerSimonian–Laird model. Leave-one-out analysis produced pooled estimates between 38.9% and 47.0%. Extending the synthesis to all 19 unselected paediatric torsion cohorts (1409 episodes) yielded a pooled proportion of 36.1% (24.1–50.0). In a formal comparison within a single model, the 5 additional cohorts had lower odds of oophorectomy than the 14 primary cohorts (odds ratio 0.34, 95% CI 0.11–1.05; *p* = 0.074); this difference was not statistically significant and is regarded as exploratory. All pooled estimates are given in Table 2.

**Figure 3:**
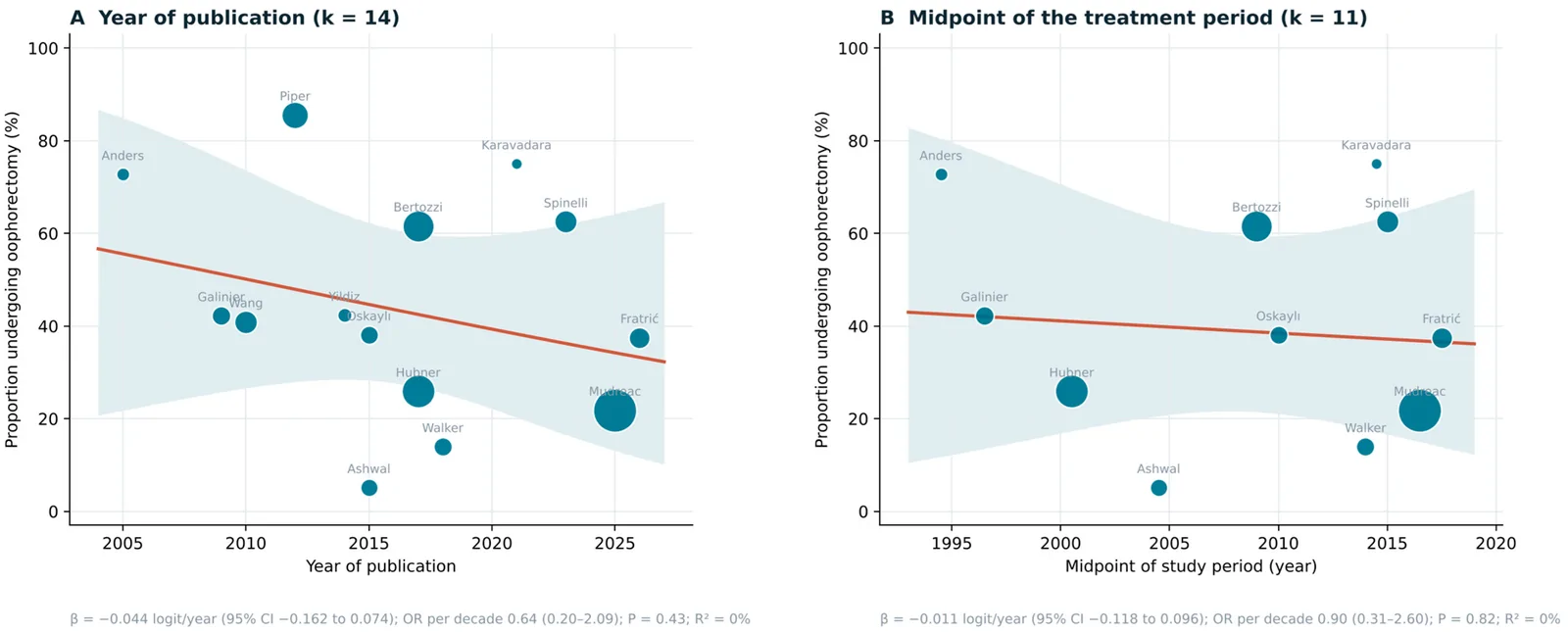
Mixed-effects meta-regression of the oophorectomy proportion on (**A**) year of publication (k = 14) and (**B**) the midpoint of the treatment period (k = 11), with fitted lines and 95% confidence bands on the proportion scale. Bubble area is proportional to cohort size. Neither moderator explained any of the between-study variance.

Direct and indirect evidence. In the four cohorts with documented premenarchal status (195 episodes), the pooled proportion was 33.4% (95% CI 8.5–69.8; prediction interval 0.2–99.3%), and in the ten indirect-evidence cohorts, it was 46.6% (29.2–65.3). The difference was not statistically significant (odds ratio 1.72, 95% CI 0.46–6.46; likelihood-ratio *p* = 0.42), but the direct-evidence estimate was very imprecise. Restricting the indirect evidence to progressively younger cohorts gave 38.9% (26.0–53.4; k = 13) at a central-age threshold of 11.0 years, 37.4% (20.7–56.7; k = 9) at 10.5 years and 34.6% (16.4–56.8; k = 7) at 10.0 years. By region, pooled proportions were 54.7% (41.0–68.9) in Europe, 42.8% (14.0–77.7) in North America and 28.4% (8.9–57.4) in Asia and the Middle East (*p* = 0.28). By publication era, the pooled proportion was 62.5% (33.9–84.8) in 2005–2012, 27.8% (11.7–50.5) in 2013–2018 and 46.9% (22.5–75.0) in 2019–2026 (*p* = 0.09). Meta-regression on year of publication showed no statistically significant association (β = −0.044 logit per year, 95% CI −0.162 to 0.074; odds ratio per decade 0.64, 95% CI 0.20–2.09; *p* = 0.43; R^2^ = 0%; residual I^2^ = 93.8%) and neither did meta-regression at the midpoint of the treatment period in the 11 cohorts that reported it (odds ratio per decade 0.90, 0.31–2.60; *p* = 0.82) (Figure 3). With 14 and 11 studies, these analyses had little power: they show that no temporal association was demonstrated, not that practice has been unchanged. Cohort size was unrelated to the oophorectomy proportion (*p* = 0.56).

### 3.4. RQ2: Imaging Findings After Ovarian-Sparing Surgery

**Figure 4:**
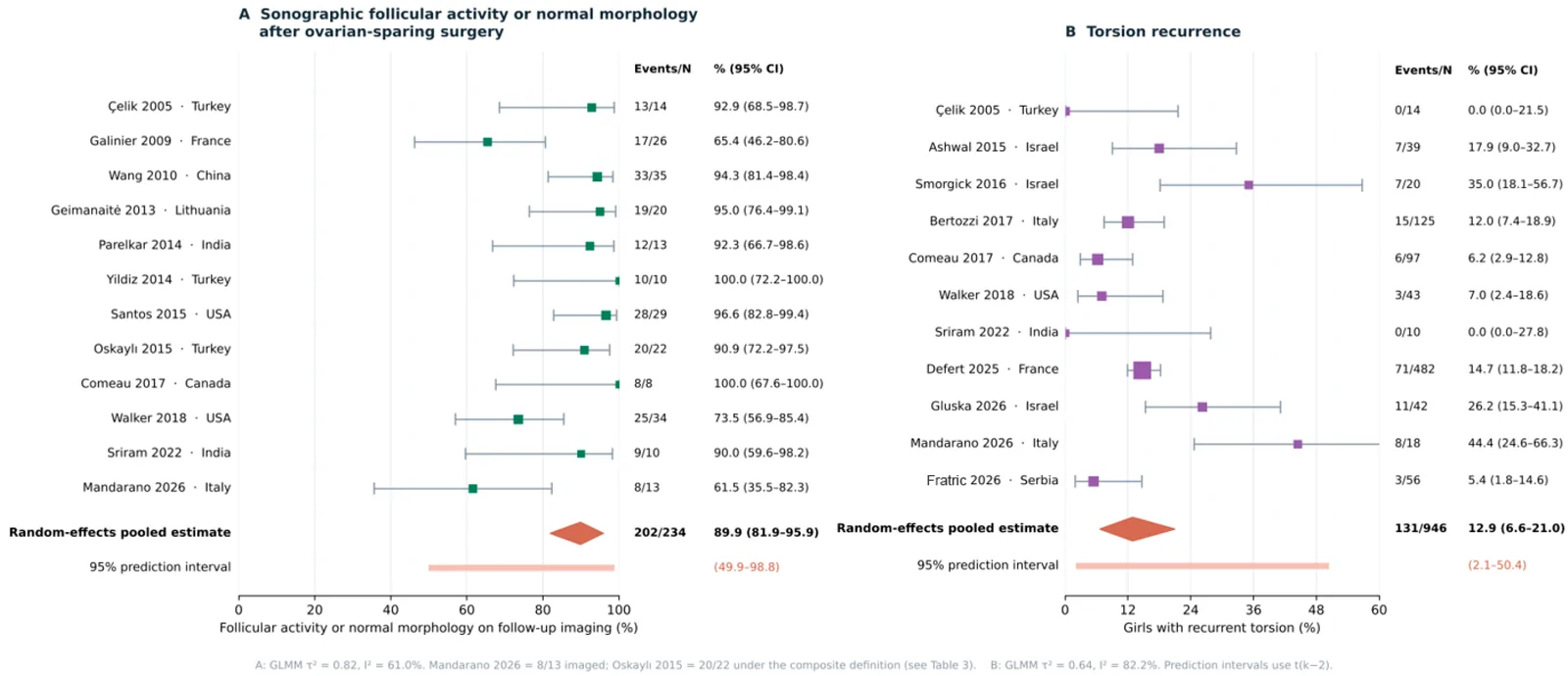
Forest plots of the secondary outcomes. (**A**) Proportion of imaged adnexa managed by ovarian-sparing surgery demonstrating follicular activity or normal morphology on follow-up ultrasound. (**B**) Proportion of girls experiencing recurrent torsion. Note the differing horizontal scales. Prediction intervals use t(k − 2).

Twelve cohorts reported follow-up imaging after detorsion with or without cystectomy or oophoropexy (Table 3). In the nine cohorts that reported both the number of adnexa conserved and the number imaged, 171 of 203 conserved adnexa (84.2%) had follow-up imaging, with completeness ranging from 65% to 100%; in the other three cohorts, the number imaged or conserved was not reported or follow-up imaging was an inclusion criterion. All cohorts used ultrasound, at least five with Doppler, at intervals ranging from three months to several years. Definitions of a favourable result differed: some studies required visible follicles, others normal size, morphology or Doppler flow, and atrophy was variably defined (Table 3). Under the composite definition of follicular activity or normal ovarian morphology, 202 of 234 imaged adnexa (crude 86.3%) met the definition, giving a pooled proportion of 89.9% (95% CI 81.9–95.9) under the generalised linear mixed model and 87.2% (77.7–93.0) under the logit model with Hartung–Knapp–Sidik–Jonkman correction. Heterogeneity was moderate to substantial (τ^2^ = 0.82; I^2^ = 61.0%; Q = 23.1, df = 11, *p* = 0.017), and the 95% prediction interval was 49.9% to 98.8% (Figure 4A). Results were similar when restricted to the nine cohorts that explicitly documented follicles (89.0%, 77.9–96.2) and to the six cohorts with documented follow-up completeness of at least 80% (89.1%, 80.3–95.8). Study-level proportions ranged from 61.5% to 100%; the lowest estimate came from the cohort with the most structured long-term imaging schedule, in which atrophy was specifically sought [21]. These are sonographic findings: no included study measured anti-Müllerian hormone, antral follicle count, endocrine function or fertility, so they cannot be read as recovery of ovarian function.

In the limited observational evidence available, the intraoperative appearance of the ovary was a poor guide to its subsequent sonographic appearance; formal diagnostic-accuracy data were not available and were not pooled. Parelkar et al. judged 10 of 13 ovaries moderately to severely ischaemic or necrotic at exploration, yet 12 of 13 showed good vascularity and follicular development on follow-up Doppler ultrasound [28]. Geimanaitė and Trainavičius conserved 31 ovaries, most of them black-blue or blue at operation, and documented multifollicular ovaries in 17 and some follicular activity in 19 of 20 girls with long-term ultrasound follow-up [27]. Sriram et al. conserved 10 consecutive ovaries that remained blue-black after detorsion and found a normal-sized ovary with preserved flow in 9 at three months [38]. Wang et al. reported normal size and follicular development in 33 of 35 premenarchal girls imaged after menarche [25].

Adverse events were not reported systematically across the included studies. Among the reports that described them, no thromboembolic event, peritonitis or sepsis attributable to leaving a detorted ovary in situ was described (Geimanaitė and Trainavičius, for example, explicitly reported none [27]); one patient developed postoperative small-bowel obstruction requiring operation after laparoscopic detorsion [34]. Absence of reported events is not equivalent to confirmed absence.

### 3.5. RQ3: Torsion Recurrence and Oophoropexy

Eleven cohorts followed 946 girls for recurrent torsion; 131 experienced at least one further episode, giving a pooled proportion of 12.9% (95% CI 6.6–21.0) under the generalised linear mixed model and 14.6% (8.3–24.4) under the logit model. Heterogeneity was substantial (τ^2^ = 0.64; I^2^ = 82.2%; Q = 34.2, df = 10, *p* < 0.001), with a 95% prediction interval of 2.1% to 50.4% (Figure 4B). The largest contributing cohort—502 prepubertal girls from 30 French paediatric surgical centres—reported recurrence in 71 of 482 girls (14.7%) who did not undergo oophoropexy at the index operation, closely matching the pooled estimate [4].

Recurrence risk was concentrated in girls whose first episode occurred before menarche and involved morphologically normal adnexa. Smorgick et al. found recurrence in 35% of premenarchal compared with 4.2% of postmenarchal patients, with premenarchal status (odds ratio 12.7, 95% CI 1.1–152.0) and torsion of normal adnexa (odds ratio 19.1, 95% CI 2.3–154.5) independently associated with recurrence [6]. Gluska et al. observed recurrence exclusively in the premenarchal group (26.2%; *p* = 0.012) [5]. These associations come from small single-centre series, and their confidence intervals are very wide, so they indicate the direction of association rather than stable risk estimates.

Evidence on oophoropexy remains observational, small and conflicting, including on the choice of fixation site. Comeau et al. performed oophoropexy in 11 of 97 girls, most often for long utero-ovarian ligaments, recurrence or bilateral torsion, with one further torsion of a pexed ovary and no procedure-related complications [33]. In Mandarano et al.’s 14-year series of primary torsion of normal adnexa (18 girls, 83% premenarchal), recurrence after the first operation was 40% after detorsion alone and 50% after detorsion with oophoropexy (*p* = 1.00); all three oophoropexies to the round ligament were followed by recurrence compared with one of five at other sites, a difference that was not statistically significant for the first operation (*p* = 0.143) and reached *p* = 0.027 only when reinterventions were added, with small numbers and some inconsistencies between the reported totals [21]. In contrast, in an adult cohort, Yagur et al. observed no recurrence after ovario–round ligament fixation (0 of 11) compared with 25.9% (7 of 27) after utero-ovarian ligament plication (*p* = 0.08) [63], and Smorgick et al. described a combined utero-ovarian and round ligament oophoropexy for recurrent torsion of normal adnexa [64]. A recent meta-analysis of paediatric primary torsion without adnexal lesions found a non-significant reduction in recurrence with oophoropexy (three comparative studies; odds ratio 0.54, 95% CI 0.17–1.72) [65]. On current evidence, no fixation site, including the round ligament, can be recommended or proscribed. No comparative study of adequate size has tested oophoropexy against detorsion alone in prepubertal girls, and these data are summarised narratively rather than pooled.

### 3.6. RQ4: Prevalence of Diagnosed Ovarian Malignancy

Seven cohorts (376 torsion episodes; one cohort reported patients rather than episodes [7]) reported the number of ovarian malignancies diagnosed. Ascertainment was incomplete and differed by surgical strategy: histology was available for removed ovaries and for excised cysts, masses or biopsies, whereas ovaries preserved without excision or biopsy had no histological verification and were followed clinically or by ultrasound (study-level diagnosed malignancy counts are listed in Supplementary File S2d). Only one cohort biopsied most conserved ovaries (10 of 15) [29]. The outcome is, therefore, diagnosed malignancy per torsion episode, not histologically excluded malignancy. Ten malignancies were diagnosed, giving a pooled prevalence of 2.7% (95% CI 1.0–4.6; exact binomial 1.3–4.8) with no detectable between-study heterogeneity (τ^2^ = 0; I^2^ = 0%; Q = 2.4, df = 6, *p* = 0.87) and a 95% prediction interval of 1.2% to 5.9%; the logit model gave 3.4% (2.2–5.4). Excluding the one cohort that listed its pathology findings without an explicit statement on malignancy [18] gave 3.0% (1.3–5.1). Three of the seven cohorts, comprising 118 episodes, reported no malignancy. Where malignancies occurred, they were predominantly low-stage: Oltmann et al. identified four malignancies among 114 patients—two serous borderline tumours, one juvenile granulosa cell tumour and one dysgerminoma, all stage I, of which three were treated by resection alone [7]—and Galinier et al. reported two low-grade malignancies among 45 cases [17]. All four pooled outcomes are summarised in Table 4.

### 3.7. Publication Bias, Methodological Quality and Sensitivity Analyses

**Figure 5:**
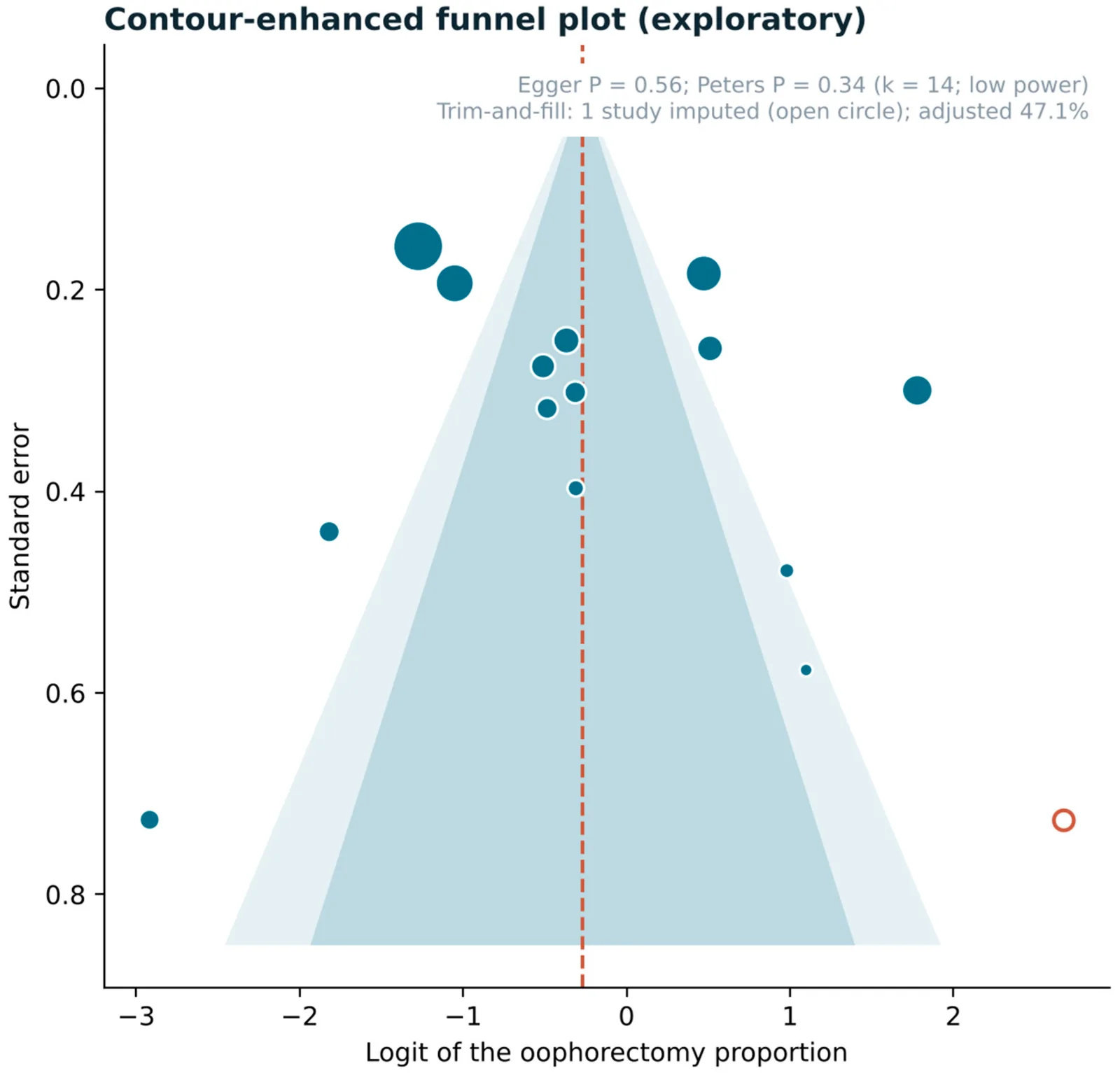
Contour-enhanced funnel plot of the logit-transformed oophorectomy proportion against its standard error, with pseudo-95% and 99% confidence limits (exploratory). Neither the Egger nor the Peters test was statistically significant; trim-and-fill imputed one study (open circle). With 14 studies these tests have low power.

Small-study effects were explored, recognising that with 14 studies, extreme heterogeneity and single proportions, these methods have low power and uncertain validity [59]. Neither the Egger regression test (t = 0.60, df = 12, *p* = 0.56) nor the Peters test (*p* = 0.34) was statistically significant; trim-and-fill imputed one study on the right of the funnel, which would raise the logit-model estimate to 47.1% (31.7–63.2) (Figure 5). These analyses cannot exclude reporting bias; institutional series may be published selectively, and outcomes such as follow-up imaging may be reported selectively.

**Figure 6:**
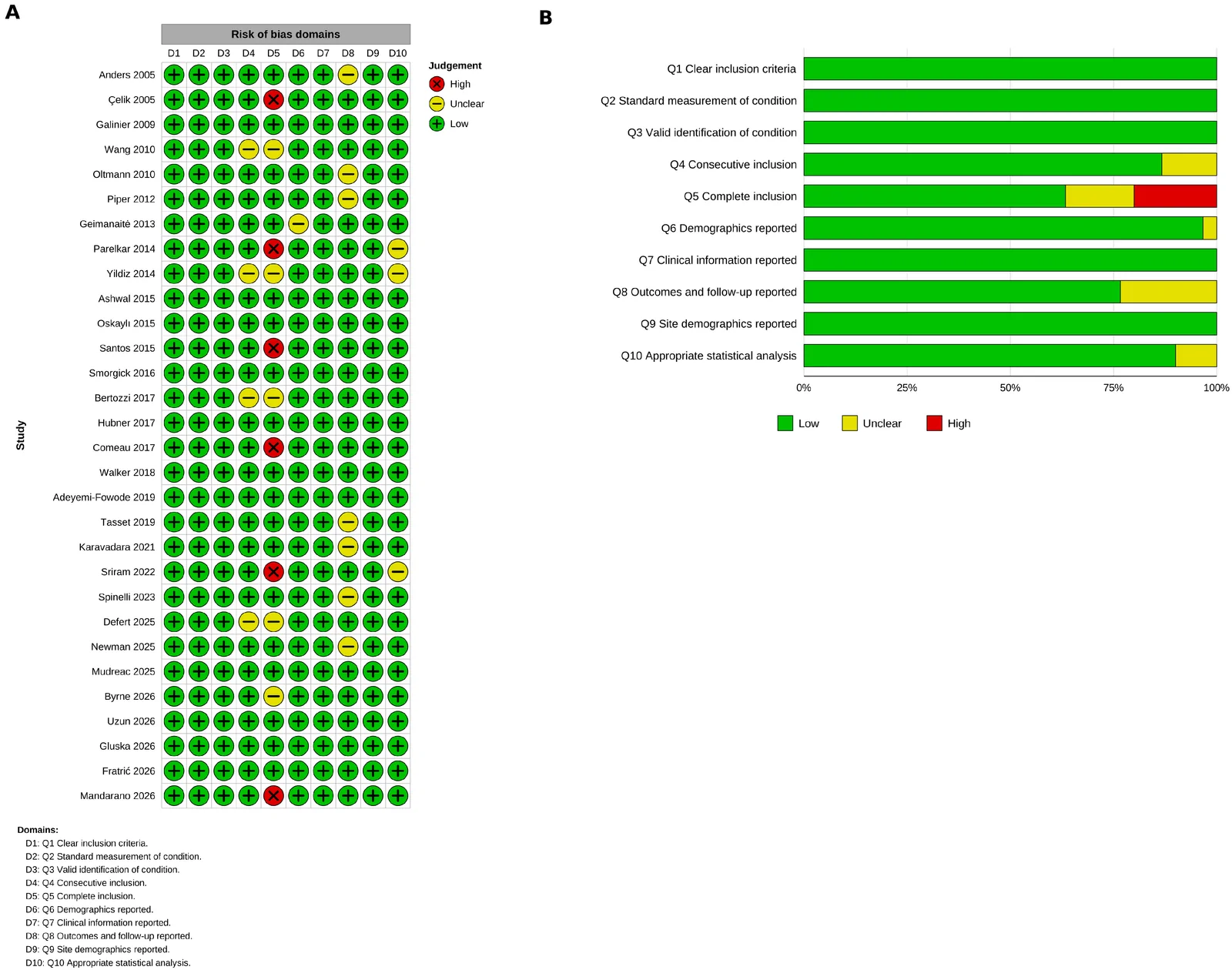
Critical appraisal of all 30 included studies using the ten-item Joanna Briggs Institute checklist for case series, shown at item level. (**A**) Traffic-light plot; (**B**) proportion of studies with each judgement for each item. A “yes” answer is plotted as low concern (green, +), “unclear” as unclear (yellow, −) and “no” as high concern (red, ×). Item judgements were not combined into an overall score.

On the JBI critical appraisal checklist, 7 of the 14 primary-analysis cohorts were rated yes on all ten items, 6 on eight or nine and 1 on seven (Figure 6). For the key domains, 3 of the 14 cohorts were rated unclear for both consecutive and complete inclusion, and 4 were rated unclear for completeness of outcome and follow-up reporting. Ascertainment of the condition was valid and standardised in every study, since inclusion required intraoperative confirmation of torsion. The items most often rated unclear were consecutive and complete inclusion of participants—typically in studies that did not state whether all eligible patients over the study period were captured—and completeness of outcome and follow-up reporting, in cohorts that recorded the operation performed but not subsequent imaging.

Restricting the synthesis to the seven cohorts rated yes on all ten JBI items (602 episodes) gave a pooled oophorectomy proportion of 24.8% (95% CI 14.4–37.3; I^2^ = 83.9%; prediction interval 5.6–64.9%), substantially lower than the main estimate (odds ratio 0.18, 95% CI 0.09–0.39, for these cohorts versus the others). The domain-based analyses were less divergent: 41.2% (23.1–61.9; k = 11) for cohorts rated yes on both selection items and 31.1% (20.1–43.7; k = 10) for cohorts rated yes on outcome reporting. The seven fully rated cohorts were on average more recently published (mean year 2018 versus 2015) and contributed 602 of the 1011 episodes, so appraisal status is not independent of era or centre volume, and with 14 studies, these explanations cannot be separated. The association, therefore, does not show that more complete documentation caused lower oophorectomy rates; it may reflect era, centre expertise, case mix or volume.

### 3.8. Exploratory Comparison of Oophorectomy and Diagnosed Malignancy

Four cohorts (176 episodes) reported both the operation performed and diagnosed malignancy on the same denominator [17,18,25,29]. Within these cohorts, 59 episodes (33.5%) ended in oophorectomy and 4 (2.3%) had a diagnosed malignancy; the crude within-cohort difference was 31.2 percentage points (cluster-bootstrap 95% interval 14.2–44.6), and a random-effects risk-difference model gave 29.3 points (95% CI 1.4–57.2; I^2^ = 86%; prediction interval −30.5 to 89.1). The difference shows that diagnosed malignancy accounted for only a small share of the oophorectomies in these cohorts. It is not an estimate of preventable ovarian loss: non-malignant indications for removal were not reported at patient level, operative and pathological data were not linked, and the outcome of removed ovaries had they been preserved is unknown. Because ovaries selected for conservation may differ systematically from those removed, the favourable imaging findings after conservation cannot be transferred to removed ovaries.

### 3.9. Population-Level Context

Seven administrative and claims datasets, which cannot separate prepubertal from adolescent patients and were, therefore, not pooled with the institutional cohorts, provide a consistent picture at population scale (Table 5). Across the five datasets reporting extractable counts, 2957 of 5612 girls and adolescents (52.7%) underwent oophorectomy. Rates fell from 78.3% in a national inpatient sample spanning 1998–2011 [66] to 22.5% in a paediatric hospital network in 2012–2017 [67], yet a national emergency department sample for 2016–2018 still reported 67.3% [68]. Two findings from these datasets bear directly on this review: children under 11 years had higher odds of oophorectomy than older children in a multicentre analysis of 1783 cases [67], and a national surgical quality database of 1791 operations for adnexal torsion found no significant difference in the proportion of oophorectomies performed before compared with after the professional guideline update (71.9% versus 69.1%; adjusted odds ratio 0.94, 95% CI 0.71–1.25) [69].

### 3.10. Certainty of the Evidence

Applying GRADE guidance for estimates of event rates [61], certainty was very low for ovarian loss (serious risk of bias, very serious inconsistency and serious indirectness) and for imaging findings after ovarian-sparing surgery (serious risk of bias, inconsistency and indirectness), and low for recurrence (serious risk of bias and inconsistency) and for diagnosed malignancy (serious risk of bias and indirectness). The full evidence profile, with the reason for every judgement, is presented in Table 6.

## 4. Discussion

### 4.1. Principal Findings

This review separates direct evidence in premenarchal girls from paediatric cohorts presumed to be predominantly prepubertal. Four findings answer the questions posed at the outset. First (RQ1), 42.8% of torsion episodes in the prepubertal-predominant evidence base ended in removal of the ovary, with extreme between-cohort variation (prediction interval 6.0–89.9%) that was not explained by era, region, cohort size or the way prepubertal status was defined; only four cohorts documented premenarchal status, and their pooled estimate (33.4%) was very imprecise. Second (RQ2), 89.9% of preserved adnexa that were imaged showed follicular activity or normal morphology, including ovaries that appeared black or blue at operation; this is a sonographic rather than a functional outcome, and imaging follow-up was incomplete. Third (RQ3), torsion recurred in 12.9% of girls, with the risk apparently concentrated in those whose first episode occurred before menarche and involved normal adnexa; the benefit of oophoropexy is unproven, and the evidence on fixation site conflicts. Fourth (RQ4), diagnosed malignancy was present in 2.7% of episodes and was typically low-stage, although preserved ovaries were not verified histologically.

Taken together, reported oophorectomy rates in many cohorts are far higher than the prevalence of diagnosed malignancy. Malignancy is not the only legitimate reason for removal, however, and the available data do not allow the proportion of avoidable oophorectomies to be quantified.

### 4.2. Comparison with Previous Work

The direction of these findings is consistent with the paediatric surgical and gynaecological literature, although these data cannot establish whether the magnitude differs in the prepubertal group. The 2018 American Pediatric Surgical Association systematic review concluded that detorsion should be standard practice in children and adolescents [9], and a recent single-arm meta-analysis of conservative laparoscopic management in paediatric and adolescent populations reported low rates of recurrence and conversion [14]. Neither analysis isolated the prepubertal group. The point estimate in the prepubertal-predominant evidence base (42.8%) was higher than in the broader paediatric analysis (36.1%), although the formal comparison was not statistically significant (*p* = 0.074). This exploratory difference is consistent with the multicentre observation that children under 11 years have higher odds of oophorectomy than older children [67]. Piper et al. reported gonadal salvage in girls (14.4%) of roughly half that achieved in boys with testicular torsion at the same institution (30.3%), which the authors attributed to slower imaging and longer times to the operating room [26]; because the biology and operative context of ovarian and testicular torsion differ, this comparison illustrates delays in the care pathway rather than equivalent salvage potential.

The imaging estimate of 89.9% is consistent with the individual cohorts on which current recommendations rest, and the lower bound of its prediction interval (49.9%) indicates that in some settings, only about half of preserved ovaries may show follicular activity or normal morphology. Long-term series suggest that atrophy becomes apparent in a minority of preserved ovaries as follow-up lengthens and as the degree of torsion increases [21,42]. Whether sonographic recovery translates into preserved ovarian reserve is less certain. In a paediatric follow-up study of 17 girls, mean anti-Müllerian hormone about six years after surgery was higher after detorsion than after oophorectomy (5.54 versus 2.70 ng/mL; *p* = 0.04), and all ten preserved ovaries appeared normal on Doppler ultrasound, although three were small for age [73]. In adults, anti-Müllerian hormone rose progressively over the six months after detorsion, with smaller recovery after longer symptom duration [74]. These small studies support, but do not establish, functional recovery; antral follicle count, endocrine outcomes and fertility after childhood torsion remain essentially unstudied.

### 4.3. Possible Explanations for Persistent Oophorectomy

No temporal association was demonstrated, despite a repeatedly restated professional consensus, although the analysis had little power and publication year is an imperfect proxy for practice era. If high oophorectomy rates do persist, three explanations are plausible and not mutually exclusive.

The first is that the problem lies upstream of the operating theatre. In prepubertal girls, torsion frequently occurs on a normal ovary, so imaging shows no mass to anchor the diagnosis, and ultrasound sensitivity in this group is imperfect [75]. By the time the abdomen is opened, the ovary has often been ischaemic for days rather than hours, and the duration of symptoms before presentation, rather than the speed of the operating-theatre pathway thereafter, is what most strongly predicts ovarian loss [41,76]. The remedy is earlier suspicion, a lower threshold for diagnostic laparoscopy when imaging is equivocal [77], and explicit recognition that a normal-appearing ovary on ultrasound does not exclude torsion.

The second is that the visual criterion itself is not trusted. In the limited observational evidence assembled here, ovarian colour at operation was a poor guide to subsequent sonographic appearance, yet it remains the dominant intraoperative cue. Changing this requires not further publication but structured intraoperative decision support and, where possible, involvement of a clinician with paediatric and adolescent gynaecology training, which has been independently associated with ovarian salvage [37].

The third is that guidelines alone do not change operative behaviour. A national surgical quality database found no significant change in oophorectomy proportions before being compared with after the guideline update [69], whereas a single institution that implemented a continuous quality improvement programme—education, collaborative care pathways and case review—raised its ovarian conservation rate from 47.6% to 85.6% [32]. This contrast between a national dataset and a single institution is hypothesis-generating rather than evidence of what works, because both sources are observational and may differ in case mix. Similarly, the cohorts rated yes on all appraisal items had lower oophorectomy proportions, but this association may reflect era, centre expertise or volume rather than any effect of documentation.

### 4.4. Clinical and Policy Implications

The pooled analyses directly support two conclusions: reported oophorectomy rates vary widely across cohorts and often exceed the prevalence of diagnosed malignancy, and most imaged preserved ovaries show follicular activity or normal morphology. These findings are consistent with existing guidance that conservation is appropriate irrespective of intraoperative appearance and that oophorectomy be reserved for an ovary that is clearly non-viable or where malignancy is strongly suspected [10]; they do not by themselves establish new recommendations. Further considerations arise from individual observational studies or from guidance rather than from the pooled estimates and carry correspondingly lower certainty. Where an underlying mass is present and malignancy is not suspected, ovarian-sparing cystectomy or tumourectomy may be considered; one series reported less retorsion after excision of an associated cyst [78], but this has not been tested comparatively. Frozen section may assist where malignancy is genuinely suspected and the service can provide it, but its availability during emergency surgery, its diagnostic limitations for paediatric ovarian tumours and the risk of prolonging the operation must be weighed [55]. Girls with torsion of normal adnexa, in whom recurrence appears more frequent [5,6], may benefit from counselling about recurrence and a low threshold for re-presentation. Oophoropexy may be considered after a recurrence, in a solitary ovary or where an elongated utero-ovarian ligament is found, but evidence on its benefit and on the choice of fixation site is conflicting [21,63,64,65]. Preservation may be impossible or unsafe when the ovary has been replaced by a mass, has autoamputated, bleeds uncontrollably or harbours a strongly suspected malignancy; when removal is performed, the indication should be documented. Follow-up of preserved ovaries, and of girls who lose an ovary, is reasonable given the endocrine as well as reproductive role of the ovary, but the optimal schedule and the role of paediatric endocrinology have not been evaluated; one multicentre cohort arranged endocrine follow-up for 30% of girls [4].

At the system level, and as a proposal by the author rather than a finding of this review, audit of ovarian conservation rates together with the documented indications for removal could make the variation documented here visible where decisions are made; a conservation rate alone, without adjustment for case mix, would be an incomplete quality indicator.

### 4.5. Strengths

To our knowledge, this is the first synthesis to separate direct evidence in premenarchal girls from paediatric cohorts presumed to be predominantly prepubertal, with an explicit eligibility definition that makes the composition of the evidence transparent. The primary model was a binomial–normal generalised linear mixed model rather than a transformation-based approximation, and three alternative models (two pre-specified) produced estimates within one percentage point of it. Prediction intervals are reported alongside confidence intervals for every synthesis, so the extent of unexplained variation is visible rather than concealed behind a single pooled figure. Every included citation was verified against its primary bibliographic record and digital object identifier, and every extracted datum was traced to the source sentence. The analysis code and study-level data are provided so that every estimate can be reproduced.

### 4.6. Limitations

Several limitations qualify these conclusions. First, screening, data extraction and appraisal were performed by a single reviewer without independent duplication, which increases the risk of missed studies and of extraction and judgement errors [19,20]; re-verification of all pooled counts during revision corrected two errors but cannot substitute for independent duplicate review. Second, this review is a restricted-source review: Embase, CINAHL, Scopus, Web of Science and Cochrane CENTRAL were not searched, and the requirement for a surgical-management concept may have reduced sensitivity, so eligible studies may have been missed. Third, all 30 studies were retrospective, so the pooled estimates inherit the selection and information biases of chart review, and no randomised or prospective comparative evidence exists in this population. Fourth, only 4 of the 14 primary cohorts documented premenarchal status; the remaining 10 were included on central age, and an estimated 21–25% of all episodes may have involved postmenarchal girls. Fifth, heterogeneity was very high for the primary outcome (I^2^ = 94.0%), and none of the subgroup analyses or meta-regressions explained it; these analyses are ecological, compare cohorts rather than patients and had little power. The estimate restricted to cohorts rated yes on all appraisal items was materially lower (24.8%). Sixth, the unit of analysis was the torsion episode in several cohorts, so girls with bilateral or recurrent torsion contribute more than once, and patients may overlap between publications: each synthesis used only one report per institution and period, but overlap between multicentre and single-centre series from the same country could not be excluded. Seventh, imaging follow-up was incomplete and possibly selective, timing varied, outcome definitions differed, and no study measured anti-Müllerian hormone, antral follicle count or fertility, so the imaging estimate may overestimate durable function. Eighth, malignancy was verified histologically only in resected or biopsied tissue, so occult lesions in preserved ovaries could have been missed. Finally, tests for small-study effects had little power, and selective publication of institutional series cannot be excluded.

### 4.7. Future Research

Three priorities follow. First, prospective multicentre registries of paediatric adnexal torsion should record menarchal status, time from symptom onset to operation, intraoperative ovarian appearance and the operation performed, so that the determinants of ovarian loss can be modelled at the level of the individual patient rather than the cohort. Second, ovarian recovery should be assessed with functional endpoints—anti-Müllerian hormone and antral follicle count at defined intervals, and ultimately fertility—rather than with ultrasound morphology alone. Third, the value of oophoropexy, and specifically the choice of fixation site, should be tested prospectively in girls with torsion of normal adnexa, the group in whom recurrence risk is highest and in whom the procedure is most often performed on the weakest evidence.

## 5. Conclusions

In prepubertal girls and paediatric cohorts presumed to be predominantly prepubertal, the reported proportion of torsion episodes managed by oophorectomy averaged 42.8% (95% CI 28.1–58.9) but varied so widely between cohorts (prediction interval 6.0–89.9%) that no single figure describes current practice; in the few cohorts with documented premenarchal status, the estimate was 33.4% and very imprecise. Most imaged ovaries preserved by ovarian-sparing surgery showed follicular activity or normal morphology (89.9%, 95% CI 81.9–95.9), and diagnosed malignancy was uncommon (2.7%, 1.0–4.6). The certainty of these estimates is low to very low. This review supports alignment with existing ovarian-conservation guidance, but it cannot quantify the proportion of oophorectomies that were avoidable. Prospective registries that record menarchal status, the documented indication for removal and functional ovarian outcomes are needed.

## Figures and Tables

**Table 1:** Characteristics of the 30 included studies.

Study [Ref]	Country	Study Period	Design	Eligibility Route	Torsion Episodes	Central Age (Years)	JBI Items Rated Yes
Anders, 2005 [23]	USA	1987–2002	Retrospective cohort	Central age ≤ 12 y	22	10.2 (mean)	9/10
Çelik, 2005 [24]	Turkey	NR	Retrospective series	Recovery/recurrence only	14	11.5 (mean)	9/10
Galinier, 2009 [17]	France	1986–2007	Retrospective cohort	Central age ≤ 12 y	45	11.0 (median)	10/10
Oltmann, 2010 [7]	USA	15.5-year review	Retrospective cohort	Malignancy only	114	10.0 (mean)	9/10
Wang, 2010 [25]	China	NR	Retrospective cohort	Premenarchal cohort	66	Premenarchal	8/10
Piper, 2012 [26]	USA	10-year review	Retrospective cohort	Central age ≤ 12 y	90	12.0 (median)	9/10
Geimanaitė, 2013 [27]	Lithuania	1989–2012	Retrospective cohort	Paediatric (sensitivity)	53	NR	9/10
Parelkar, 2014 [28]	India	2009–2013	Retrospective series	Recovery only	13	NR	8/10
Yildiz, 2014 [29]	Turkey	NR	Retrospective cohort	Premenarchal cohort	26	Premenarchal	7/10
Ashwal, 2015 [18]	Israel	1997–2012	Retrospective cohort	Premenarchal cohort	39	9.0 (median)	10/10
Oskaylı, 2015 [22]	Turkey	2006–2014	Retrospective cohort	Central age ≤ 12 y	42	11.0 (mean)	10/10
Santos, 2015 [30]	USA	2007–2010	Retrospective series	Recovery only	29	10.3 (mean)	9/10
Smorgick, 2016 [6]	Israel	NR	Retrospective cohort	Recurrence only	20	Premenarchal	10/10
Bertozzi, 2017 [31]	Italy	2004–2014	Multicentre retrospective	Central age ≤ 12 y	125	9.8 (mean)	8/10
Hubner, 2017 [32]	Canada	1988–2013	Retrospective before–after	Central age ≤ 12 y	139	10.2 (mean)	10/10
Comeau, 2017 [33]	Canada	2004–2013	Retrospective series	Recovery/recurrence only	97	8.8 (OP subgroup)	9/10
Walker, 2018 [34]	USA	2012–2016	Retrospective cohort	Central age ≤ 12 y	43	8.3 (median)	10/10
Adeyemi-Fowode, 2019 [35]	USA	2007–2016	Retrospective cohort	Paediatric (sensitivity)	245	12.4 (mean)	10/10
Tasset, 2019 [36]	USA	2006–2017	Retrospective case–control	Malignancy only	33	0–12 (range)	9/10
Karavadara, 2021 [37]	UK	2011–2018	Retrospective cohort	Central age ≤ 12 y	16	11.0 (median)	9/10
Sriram, 2022 [38]	India	2016–2021	Retrospective series	Recovery/recurrence only	10	11.0 (mean)	8/10
Spinelli, 2023 [15]	Italy/Germany	2010–2020	Multicentre retrospective	Premenarchal subgroup	64	≤12 (subgroup)	9/10
Defert, 2025 [4]	France	2000–2022	Multicentre retrospective	Recurrence only	482	7.9 (mean)	8/10
Newman, 2025 [39]	UK	2016–2024	Retrospective cohort	Paediatric (sensitivity)	23	NR (<16 y)	9/10
Mudreac, 2025 [40]	USA	2010–2023	Retrospective cohort	Central age ≤ 12 y	238	10.9 (mean)	10/10
Byrne, 2026 [41]	USA	2015–2024	Retrospective series	Paediatric (sensitivity)	34	NR (1–17 y)	9/10
Uzun, 2026 [42]	Turkey	2019–2022	Retrospective cohort	Paediatric (sensitivity)	43	NR (0–18 y)	10/10
Gluska, 2026 [5]	Israel	2016–2023	Retrospective cohort	Recurrence only	42	Premenarchal	10/10
Fratrić, 2026 [43]	Serbia	2010–2025	Retrospective cohort	Central age ≤ 12 y	56	10.0 (median)	10/10
Mandarano, 2026 [21]	Italy	2010–2024	Retrospective series	Recovery/recurrence only	18	9.4 (median)	9/10

**Table 2:** Pooled proportion of torsion episodes managed by oophorectomy: primary model, alternative models, direct and indirect evidence, sensitivity analyses and subgroups.

Analysis	k	Episodes	Events	Pooled % (95% CI)	I^2^ (%)	τ^2^	95% PI (%)
Primary: binomial–normal GLMM, all cohorts	14	1011	412	42.8 (28.1–58.9)	94.0	1.19	6.0–89.9
Logit, REML, HKSJ correction	14	1011	412	43.3 (27.8–60.2)	93.9	1.17	6.1–89.9
Freeman–Tukey double arcsine	14	1011	412	43.4 (30.1–57.3)	94.5	0.06 †	3.6–89.4
DerSimonian–Laird, no HKSJ	14	1011	412	43.4 (31.0–56.6)	92.2	0.90	10.0–84.2
Direct evidence: premenarchal status documented	4	195	80	33.4 (8.5–69.8)	91.9	1.31	0.2–99.3
Indirect evidence: central age ≤ 12.0 y	10	816	332	46.6 (29.2–65.3)	94.1	1.11	6.3–91.8
Threshold: direct + central age ≤ 11.0 y	13	921	335	38.9 (26.0–53.4)	92.0	0.87	7.0–84.4
Threshold: direct + central age ≤ 10.5 y	9	580	236	37.4 (20.7–56.7)	92.8	1.05	4.4–88.6
Threshold: direct + central age ≤ 10.0 y	7	419	184	34.6 (16.4–56.8)	92.3	1.05	3.0–90.1
Sensitivity: all paediatric cohorts	19	1409	472	36.1 (24.1–50.0)	94.1	1.32	4.4–87.3
Sensitivity: JBI all ten items yes	7	602	152	24.8 (14.4–37.3)	83.9	0.38	5.6–64.9
Sensitivity: selection items 4 and 5 yes ‡	11	794	297	41.2 (23.1–61.9)	94.8	1.54	3.6–93.0
Sensitivity: outcome item 8 yes ‡	10	819	267	31.1 (20.1–43.7)	89.3	0.55	6.9–73.4
Era: 2005–2012	4	223	139	62.5 (33.9–84.8)	88.7	0.78	2.2–99.2
Era: 2013–2018	6	414	148	27.8 (11.7–50.5)	91.8	0.97	1.9–88.4
Era: 2019–2026	4	374	125	46.9 (22.5–75.0)	91.1	0.72	1.4–98.2
Region: North America	5	532	187	42.8 (14.0–77.7)	96.6	1.91	0.6–99.0
Region: Europe	5	306	169	54.7 (41.0–68.9)	69.9	0.18	20.6–84.8
Region: Asia and Middle East	4	173	56	28.4 (8.9–57.4)	85.2	0.77	0.5–96.8

k, number of studies; CI, confidence interval; PI, prediction interval; τ^2^, between-study variance on the logit scale; GLMM, generalised linear mixed model; REML, restricted maximum likelihood; HKSJ, Hartung–Knapp–Sidik–Jonkman. Unless stated otherwise, estimates are from the GLMM fitted with adaptive Gauss–Hermite quadrature, with profile-likelihood confidence intervals; prediction intervals are $\hat{µ}\pm t\left(k-2\right)\times \surd \left({\hat{\tau }}^{2}+{\mathrm{SE}}^{2}\right)$ and are very wide when k is small. † On the double-arcsine scale. ‡ Domain-based sensitivity analyses added post hoc at revision. Between-group comparisons (GLMM likelihood-ratio tests): direct versus indirect evidence, odds ratio 1.72 (95% CI 0.46–6.46), *p* = 0.42; all paediatric versus primary cohorts, 0.34 (0.11–1.05), *p* = 0.074; JBI all items versus not, 0.18 (0.09–0.39), *p* < 0.001; era, *p* = 0.09; region, *p* = 0.28.

**Table 3:** Follow-up imaging after ovarian-sparing surgery: completeness, modality, timing and outcome definitions.

Atrophy or Abnormal (n)	Met Definition (n)	Favourable Result as Defined	Timing	Modality	Imaged, n (%)	Conserved (n)	Study [ref]
1 atrophic	13	Normal size and folliculogenesis	3–66 mo (mean 21.9)	Colour Doppler US	14 (100)	14	Çelik, 2005 [24]
NR	17	Follicular ovary	Several months	US	NR	26	Galinier, 2009 [17]
2 atrophic	33	Normal size and follicles	After menarche	US	35 (90)	39	Wang, 2010 [25]
1 ovary not detected	19	Any follicles (17 multifollicular)	Long-term, NR	US	20 (65)	31	Geimanaitė, 2013 [27]
1 without follicles	12	Vascularity and follicles	NR	Colour Doppler US	13 (100)	13	Parelkar, 2014 [28]
0	10	Normal follicular development	6 mo	Doppler US	10 (67)	15	Yildiz, 2014 [29]
NR	28	Follicles on affected side	Mean 8.1 mo	US	29	NR *	Santos, 2015 [30]
5 smaller, 2 without follicles	20	Normal size and flow (17) or follicles (3)	Mean 25 mo	Colour Doppler US	22 (85)	26	Oskaylı, 2015 [22]
NR	8	≥1 marker of normal function	NR	US	8	NR	Comeau, 2017 [33]
NR	25	Follicles visualised	NR	NR	34 (92)	37	Walker, 2018 [34]
1 very small	9	Normal size and flow	3 mo	Doppler US	10 (100)	10	Sriram, 2022 [38]
5 hypotrophic or afollicular	8	Normal size and follicles	1, 3, 12 mo, then yearly	US	13 (72)	18	Mandarano, 2026 [21]
	202 of 234				171 (84.2) †	203 †	Total

US, ultrasound; NR, not reported. Data were extracted from the published abstract or full text. * Follow-up ultrasound was an inclusion criterion. For Galinier et al. the number imaged was not reported, and all 26 conserved adnexa were used as the denominator. † Totals for the nine cohorts reporting both the number conserved and the number imaged. The composite outcome counted an adnexa as favourable if follicles or normal size or morphology were documented; component-level data are in Supplementary File S2b.

**Table 4:** Summary of all four pooled outcomes and their certainty.

Certainty (GRADE)	95% PI (%)	τ^2^	I^2^ (%)	Pooled % (95% CI)	Events	Denominator	k	Outcome
Very low	6.0–89.9	1.19	94	42.8 (28.1–58.9)	412	1011 episodes	14	Ovarian loss (oophorectomy, salpingo-oophorectomy or adnexectomy), all primary cohorts
—	0.2–99.3	1.31	91.9	33.4 (8.5–69.8)	80	195 episodes	4	Direct evidence only (premenarchal status documented)
Very low	49.9–98.8	0.82	61	89.9 (81.9–95.9)	202	234 adnexa imaged	12	Follicular activity or normal morphology on imaging after ovarian-sparing surgery
Low	2.1–50.4	0.64	82.2	12.9 (6.6–21.0)	131	946 girls	11	Recurrent torsion
Low	1.2–5.9	0	0	2.7 (1.0–4.6)	10	376 episodes	7	Diagnosed ovarian malignancy

All estimates are from the binomial–normal generalised linear mixed model with profile-likelihood confidence intervals; prediction intervals use t(k − 2). Denominators are torsion episodes for ovarian loss and malignancy, imaged adnexa for imaging findings, and girls for recurrence. Malignancy was verified histologically only in resected or biopsied tissue. Certainty ratings are explained in Section 3.10.

**Table 5:** Population-level and administrative datasets (contextual; not pooled with the institutional cohorts).

Key Observation	Oophorectomy n (%)	n	Age Range	Source and Period	Country	Dataset [Ref]
Incidence 4.9/100,000	713 (57.9)	1232	1–20 y	Kids’ Inpatient Database 2006	USA	Guthrie, 2010 [1]
Conservation rose 9% → 25%	1598 (78.3)	2041	<18 y	Nationwide Inpatient Sample 1998–2011	USA	Sola, 2015 [66]
38% (surgeons) vs. 27% (gynaecologists)	Not reported as count	1151	Mean 10.7 y	PHIS 2007–2011	USA	Campbell, 2015 [70]
Higher odds if aged <11 y	402 (22.5)	1783	<18 y	PHIS 2012–2017	USA	Lipsett, 2021 [67]
Rate fell over time	Not reported as count	7008	1–20 y	Kids’ Inpatient Database 2003–2019	USA	Ayemoba, 2024 [71]
48% minimally invasive	177 (67.3)	263	<20 y	Nationwide Emergency Dept Sample 2016–2018	USA	Das, 2025 [68]
Open oophorectomy 26% vs. 4% by unit type	67 (22.9)	293	Mean 12.4 y	Statutory health insurance claims 2010–2019	Germany	Feng, 2026 [72]

PHIS, Pediatric Health Information System. These datasets identify patients by administrative code and cannot distinguish prepubertal from postmenarchal girls; they are presented for context only and were not combined with the institutional cohorts in any meta-analysis.

**Table 6:** GRADE evidence profile.

Certainty	Publication Bias	Imprecision	Indirectness	Inconsistency	Risk of Bias	Studies (Denominator)	Outcome
⨁◯◯◯ Very low	Not rated down e	Not serious d	Serious c	Very serious b	Serious a	14 obs. (1011 episodes)	Ovarian loss
⨁◯◯◯ Very low	Not rated down e	Not serious	Serious h	Serious g	Serious f	12 obs. (234 adnexa)	Follicular activity or normal morphology on imaging
⨁⨁◯◯ Low	Not rated down e	Not serious	Not serious k	Serious j	Serious i	11 obs. (946 girls)	Recurrent torsion
⨁⨁◯◯ Low	Not rated down e	Not serious n	Serious m	Not serious	Serious l	7 obs. (376 episodes)	Diagnosed malignancy

obs., observational studies. Following GRADE guidance for event rates [61], each body of evidence started at high certainty and was rated down. a Retrospective series; consecutive and complete inclusion unclear in 3 of 14 cohorts; the estimate fell to 24.8% in the 7 cohorts rated yes on all appraisal items. b Prediction interval 6.0–89.9% (span 84 points). c 816 of 1011 episodes (81%) from cohorts without documented premenarchal status; an estimated 21–25% of all episodes postmenarchal. d Confidence interval wide, but this uncertainty is already captured by the inconsistency rating. e Statistical tests underpowered and not used as evidence of absence; selective publication of institutional series and selective outcome reporting are possible, so publication bias cannot be excluded. f Imaging available for 84.2% of conserved adnexa where reported; denominator unreported or imaging an inclusion criterion in 3 cohorts; variable timing. g Prediction interval 49.9–98.8% (span 49 points). h Sonographic surrogate rather than functional outcome; 189 of 234 imaged adnexa (81%) from cohorts without documented premenarchal status. i Duration and completeness of follow-up variable and often unreported. j Prediction interval 2.1–50.4% (span 48 points). k 583 of 946 girls (62%) from cohorts with documented premenarchal or prepubertal status. l Preserved ovaries without excision or biopsy had no histological verification. m 212 of 376 episodes (56%) from cohorts without documented premenarchal status. n Both confidence limits (1.0–4.6%) indicate a low prevalence, although based on only 10 events. ⨁ degree of certainty.

## Data Availability

The available study-level extraction data, item-level critical appraisal judgements, pooled statistical summaries and recorded study-selection decisions are provided in Supplementary Files S2 and S3. Original executable analysis scripts and R session information are not included in the Supplementary Materials supplied with this version.

## Supplementary Materials

The following supporting information can be downloaded at: https://www.mdpi.com/article/10.3390/jcm15218678/s1. Supplementary File S1 (PDF) contains the database-specific search strategies and the completed PRISMA 2020 checklist (File S4). Supplementary File S2 (XLSX) contains S2a, ovarian loss; S2b, ovarian recovery; S2c, recurrence; S2d, malignancy; S2e, broader paediatric sensitivity cohorts; S2f, administrative datasets; S2g, item-level JBI appraisal; and S2h, pooled statistical results. The same spreadsheet contains File S3, the available screening and eligibility log.

## Abbreviations

The following abbreviations are used in this manuscript:

CI	confidence interval
GLMM	generalised linear mixed model
GRADE	Grading of Recommendations Assessment, Development and Evaluation
HKSJ	Hartung–Knapp–Sidik–Jonkman
JBI	Joanna Briggs Institute
PI	prediction interval
PRISMA	Preferred Reporting Items for Systematic Reviews and Meta-Analyses
REML	restricted maximum likelihood

## References

[1] Guthrie B.D., Adler M.D., Powell E.C. (2010). Incidence and trends of pediatric ovarian torsion hospitalizations in the United States, 2000–2006. Pediatrics.

[2] Childress K.J., Dietrich J.E. (2017). Pediatric ovarian torsion. Surg. Clin. N. Am..

[3] Spinelli C., Piscioneri J., Strambi S. (2015). Adnexal torsion in adolescents: Update and review of the literature. Curr. Opin. Obstet. Gynecol..

[4] Defert C., Cartault A., Haffreingue A., Clermidi P., Lefébure C., Olland A., Glenisson M., Deleforterie T., Bourg A., Vérot P.-L. (2025). Ovarian torsion in prepubertal girls: Clinical characteristics, biological and radiological features, recurrency rate. J. Pediatr. Surg..

[5] Gluska H., Lidor I., Manor Bar C., Yagur Y., Zvi K., Yair D., Arbib N. (2026). Don’t hesitate, operate: Clinical implications of adnexal torsion in premenarchal girls. J. Minim. Invasive Gynecol..

[6] Smorgick N., Melcer Y., Sarig-Meth T., Maymon R., Vaknin Z., Pansky M. (2016). High risk of recurrent torsion in premenarchal girls with torsion of normal adnexa. Fertil. Steril..

[7] Oltmann S.C., Fischer A., Barber R., Huang R., Hicks B., Garcia N. (2010). Pediatric ovarian malignancy presenting as ovarian torsion: Incidence and relevance. J. Pediatr. Surg..

[8] Nur Azurah A.G., Zainol Z.W., Zainuddin A.A., Lim P.S., Sulaiman A.S., Ng B.K. (2015). Update on the management of ovarian torsion in children and adolescents. World J. Pediatr..

[9] Dasgupta R., Renaud E., Goldin A.B., Baird R., Cameron D.B., Arnold M.A., Diefenbach K.A., Gosain A., Grabowski J., Guner Y.S. (2018). Ovarian torsion in pediatric and adolescent patients: A systematic review. J. Pediatr. Surg..

[10] American College of Obstetricians and Gynecologists (2019). Adnexal torsion in adolescents: ACOG Committee Opinion No. 783. Obstet. Gynecol..

[11] Al-Turki H.A. (2015). Fertility after oophorectomy due to torsion. Saudi Med. J..

[12] Lind T., Nilsson H.P., Franko M.A., Rodriguez-Wallberg K.A. (2026). Likelihood of childbirth in women with one versus two ovaries: A Swedish population-based study. Hum. Reprod. Open.

[13] Mayhew A.C., Bost J., Linam L., Milla S., Farahzad M., Childress K.J. (2020). Compensatory ovarian hypertrophy after unilateral oophorectomy: Evaluation of ovarian volume. J. Pediatr. Adolesc. Gynecol..

[14] Marchand G.J., Massoud A., Arroyo A., González D.H., Hamilton B., Ruffley K., Robinson M., Dominick M., Ulibarri H. (2024). Efficacy of conservative laparoscopic surgical treatment for acute ovarian torsion in pediatric and adolescent populations: A single-armed meta-analysis. Turk. J. Obstet. Gynecol..

[15] Spinelli C., Tröbs R.B., Nissen M., Strambi S., Ghionzoli M., Bertocchini A., Domass V.C., Sanna B., Morganti R., Molinaro F. (2023). Ovarian torsion in the pediatric population: Predictive factors for ovarian-sparing surgery—An international retrospective multicenter study and a systematic review. Arch. Gynecol. Obstet..

[16] Page M.J., McKenzie J.E., Bossuyt P.M., Boutron I., Hoffmann T.C., Mulrow C.D., Shamseer L., Tetzlaff J.M., Akl E.A., Brennan S.E. (2021). The PRISMA 2020 statement: An updated guideline for reporting systematic reviews. BMJ.

[17] Galinier P., Carfagna L., Delsol M., Ballouhey Q., Lemasson F., Le Mandat A., Moscovici J., Guitard J., Pienkowski C., Vaysse P. (2009). Ovarian torsion. Management and ovarian prognosis: A report of 45 cases. J. Pediatr. Surg..

[18] Ashwal E., Krissi H., Hiersch L., Less S., Eitan R., Peled Y. (2015). Presentation, diagnosis, and treatment of ovarian torsion in premenarchal girls. J. Pediatr. Adolesc. Gynecol..

[19] Waffenschmidt S., Knelangen M., Sieben W., Bühn S., Pieper D. (2019). Single screening versus conventional double screening for study selection in systematic reviews: A methodological systematic review. BMC Med. Res. Methodol..

[20] Buscemi N., Hartling L., Vandermeer B., Tjosvold L., Klassen T.P. (2006). Single data extraction generated more errors than double data extraction in systematic reviews. J. Clin. Epidemiol..

[21] Mandarano G., Parolini F., Bosisio M., Gramaglia S., Prato D.M.D.P., Boroni G., Stegher C., Odicino F.E., Alberti D. (2026). Primary ovarian torsion in pediatric patients: Is oophoropexy still an option? A 14-year single-center experience. J. Pediatr. Adolesc. Gynecol..

[22] Oskaylı M.Ç., Durakbaşa Ç.U., Maşrabacı K., Mutuş H.M., Zemheri I.E., Okur H. (2015). Surgical approach to ovarian torsion in children. J. Pediatr. Adolesc. Gynecol..

[23] Anders J.F., Powell E.C. (2005). Urgency of evaluation and outcome of acute ovarian torsion in pediatric patients. Arch. Pediatr. Adolesc. Med..

[24] Çelik A., Ergün O., Aldemir H., Özcan C., Özok G., Erdener A., Balýk E. (2005). Long-term results of conservative management of adnexal torsion in children. J. Pediatr. Surg..

[25] Wang J.H., Wu D.H., Jin H., Wu Y.Z. (2010). Predominant etiology of adnexal torsion and ovarian outcome after detorsion in premenarchal girls. Eur. J. Pediatr. Surg..

[26] Piper H.G., Oltmann S.C., Xu L., Adusumilli S., Fischer A.C. (2012). Ovarian torsion: Diagnosis of inclusion mandates earlier intervention. J. Pediatr. Surg..

[27] Geimanaite L., Trainavicius K. (2013). Ovarian torsion in children: Management and outcomes. J. Pediatr. Surg..

[28] Parelkar S.V., Mundada D., Sanghvi B.V., Joshi P.B., Oak S.N., Kapadnis S.P., Shetty S., Athawale H., Multani P. (2014). Should the ovary always be conserved in torsion? A tertiary care institute experience. J. Pediatr. Surg..

[29] Yildiz A., Erginel B., Akin M., Karadağ C., Sever N., Tanik C., Canmemiş A., Dokucu A. (2014). A retrospective review of the adnexal outcome after detorsion in premenarchal girls. Afr. J. Paediatr. Surg..

[30] Santos X.M., Cass D.L., Dietrich J.E. (2015). Outcome following detorsion of torsed adnexa in children. J. Pediatr. Adolesc. Gynecol..

[31] Bertozzi M., Esposito C., Vella C., Briganti V., Zampieri N., Codrich D., Ubertazzi M., Trucchi A., Magrini E., Battaglia S. (2017). Pediatric ovarian torsion and its recurrence: A multicenter study. J. Pediatr. Adolesc. Gynecol..

[32] Hubner N., Langer J.C., Kives S., Allen L.M. (2017). Evolution in the management of pediatric and adolescent ovarian torsion as a result of quality improvement measures. J. Pediatr. Adolesc. Gynecol..

[33] Comeau I.M., Hubner N., Kives S.L., Allen L.M. (2017). Rates and technique for oophoropexy in pediatric ovarian torsion: A single-institution case series. J. Pediatr. Adolesc. Gynecol..

[34] Walker S.K., Lal D.R., Boyd K.P., Sato T.T. (2018). Management of pediatric ovarian torsion: Evidence of follicular development after ovarian preservation. Surgery.

[35] Adeyemi-Fowode O., Lin E.G., Syed F., Sangi-Haghpeykar H., Zhu H., Dietrich J.E. (2019). Adnexal torsion in children and adolescents: A retrospective review of 245 cases at a single institution. J. Pediatr. Adolesc. Gynecol..

[36] Tasset J., Rosen M.W., Bell S., Smith Y.R., Quint E.H. (2019). Ovarian torsion in premenarchal girls. J. Pediatr. Adolesc. Gynecol..

[37] Karavadara D., Davidson J.R., Story L., Diab Y., Upadhyaya M. (2021). Missed opportunities for ovarian salvage in children: An 8-year review of surgically managed ovarian lesions at a tertiary pediatric surgery centre. Pediatr. Surg. Int..

[38] Sriram R., Zameer M.M., Vinay C., Giridhar B.S. (2022). Black ovary: Our experience with oophoropexy in all cases of pediatric ovarian torsion and review of relevant literature. J. Indian Assoc. Pediatr. Surg..

[39] Newman M., Smith T., Hammond P. (2025). Paediatric and adolescent ovarian torsion: An eight-year retrospective cohort study and literature review. Acta Chir. Belg..

[40] Mudreac A., Kim S.E., Hwang R., Mattei P. (2025). Ovarian salvage following adnexal torsion in pediatric patients. Pediatr. Surg. Int..

[41] Byrne M.M., Loszko A., Wirth K., Nicandri K., Arca M.J. (2026). Time’s a tickin’: When should surgical intervention occur in ovarian torsion?. J. Pediatr. Surg..

[42] Uzun A.G.K., Senel E. (2026). Long-term ovarian function, follicular development, and atrophy after pediatric ovarian torsion. Pediatr. Surg. Int..

[43] Fratrić I., Jokić R., Antić J., Bodonji S., Pajić M. (2026). Clinical characteristics and surgical outcomes of pediatric ovarian torsion: A 15-year single-center experience. Children.

[44] Munn Z., Barker T.H., Moola S., Tufanaru C., Stern C., McArthur A., Stephenson M., Aromataris E. (2020). Methodological quality of case series studies: An introduction to the JBI critical appraisal tool. JBI Evid. Synth..

[45] Jüni P., Witschi A., Bloch R., Egger M. (1999). The hazards of scoring the quality of clinical trials for meta-analysis. JAMA.

[46] Stijnen T., Hamza T.H., Özdemir P. (2010). Random effects meta-analysis of event outcome in the framework of the generalized linear mixed model with applications in sparse data. Stat. Med..

[47] Viechtbauer W. (2010). Conducting meta-analyses in R with the metafor package. J. Stat. Softw..

[48] Bates D., Mächler M., Bolker B., Walker S. (2015). Fitting linear mixed-effects models using lme4. J. Stat. Softw..

[49] Hartung J., Knapp G. (2001). A refined method for the meta-analysis of controlled clinical trials with binary outcome. Stat. Med..

[50] Barendregt J.J., Doi S.A., Lee Y.Y., Norman R.E., Vos T. (2013). Meta-analysis of prevalence. J. Epidemiol. Community Health.

[51] DerSimonian R., Laird N. (1986). Meta-analysis in clinical trials. Control Clin. Trials.

[52] Higgins J.P.T., Thompson S.G. (2002). Quantifying heterogeneity in a meta-analysis. Stat. Med..

[53] IntHout J., Ioannidis J.P.A., Rovers M.M., Goeman J.J. (2016). Plea for routinely presenting prediction intervals in meta-analysis. BMJ Open.

[54] Higgins J.P.T., Thompson S.G., Spiegelhalter D.J. (2009). A re-evaluation of random-effects meta-analysis. J. R Stat. Soc. Ser. A Stat. Soc..

[55] Chumlea W.C., Schubert C.M., Roche A.F., Kulin H.E., Lee P.A., Himes J.H., Sun S.S. (2003). Age at menarche and racial comparisons in US girls. Pediatrics.

[56] Egger M., Davey Smith G., Schneider M., Minder C. (1997). Bias in meta-analysis detected by a simple, graphical test. BMJ.

[57] Peters J.L., Sutton A.J., Jones D.R., Abrams K.R., Rushton L. (2006). Comparison of two methods to detect publication bias in meta-analysis. JAMA.

[58] Duval S., Tweedie R. (2000). Trim and fill: A simple funnel-plot-based method of testing and adjusting for publication bias in meta-analysis. Biometrics.

[59] Hunter J.P., Saratzis A., Sutton A.J., Boucher R.H., Sayers R.D., Bown M.J. (2014). In meta-analyses of proportion studies, funnel plots were found to be an inaccurate method of assessing publication bias. J. Clin. Epidemiol..

[60] Guyatt G., Oxman A.D., Akl E.A., Kunz R., Vist G., Brozek J., Norris S., Falck-Ytter Y., Glasziou P., DeBeer H. (2011). GRADE guidelines: 1. Introduction—GRADE evidence profiles and summary of findings tables. J. Clin. Epidemiol..

[61] Iorio A., Spencer F.A., Falavigna M., Alba C., Lang E., Burnand B., McGinn T., Hayden J., Williams K., Shea B. (2015). Use of GRADE for assessment of evidence about prognosis: Rating confidence in estimates of event rates in broad categories of patients. BMJ.

[62] Guyatt G.H., Oxman A.D., Kunz R., Woodcock J., Brozek J., Helfand M., Alonso-Coello P., Falck-Ytter Y., Jaeschke R., Vist G. (2011). GRADE guidelines: 8. Rating the quality of evidence—Indirectness. J. Clin. Epidemiol..

[63] Yagur Y., Klein A., Tiosano L., Weitzner O., Daykan Y., Rosen D., Klein Z., Schonman R. (2025). Comparative outcomes of oophoropexy techniques in preventing recurrent adnexal torsion. Arch. Gynecol. Obstet..

[64] Smorgick N., Mor M., Dovev M.N., Eisenberg N., Vaknin Z. (2023). Combined utero-ovarian and round ligament oophoropexy for recurrent torsion of normal adnexa. J. Pediatr. Adolesc. Gynecol..

[65] Mandarano G., Gazzaneo M., Bosisio M., Parolini F., De Previde Prato D., Boroni G., Alberti D. (2026). Role of oophoropexy in pediatric primary ovarian torsion without adnexal lesions: A systematic review and meta-analysis. J. Pediatr. Surg..

[66] Sola R., Wormer B.A., Walters A.L., Heniford B.T., Schulman A.M. (2015). National trends in the surgical treatment of ovarian torsion in children: An analysis of 2041 pediatric patients utilizing the Nationwide Inpatient Sample. Am. Surg..

[67] Lipsett S.C., Haines L., Monuteaux M.C., Hayes K., Michelson K.A. (2021). Variation in oophorectomy rates for children with ovarian torsion across US children’s hospitals. J. Pediatr..

[68] Das K.J.H., Huynh V., Wang S., Trujillo Rivera E.A., Gomez Lobo V. (2025). Oophorectomy rates in pediatric and adolescent patients with adnexal torsion in US emergency departments. J. Pediatr. Adolesc. Gynecol..

[69] Ryles H.T., Hong C.X., Andy U.U., Farrow M.R. (2023). Changing practices in the surgical management of adnexal torsion: An analysis of the National Surgical Quality Improvement Program database. Obstet. Gynecol..

[70] Campbell B.T., Austin D.M., Kahn O., McCann M.C., Lerer T.J., Lee K., Thaker S., Herbst K.W., Rader C.M. (2015). Current trends in the surgical treatment of pediatric ovarian torsion: We can do better. J. Pediatr. Surg..

[71] Ayemoba J., Callier K., Johnson K. (2024). Rate of oophorectomy in pediatric ovarian torsion: Risk factors and change over time. Pediatr. Surg. Int..

[72] Feng X., Zimmermann P., Pardey N., Gnatzy R., Bassler S., Stahmeyer J.T., Lacher M., Zeidler J. (2026). Preservation vs. resection? Pediatric and non-pediatric management patterns in ovarian torsion. Pediatr. Rep..

[73] Oskayli M.C., Gulcin N., Ozatman E., Gercel G., Mutus M., Aksu B., Durakbasa C.U. (2019). Assessment of ovarian reserve using serum anti-Müllerian hormone after ovarian torsion surgery. Pediatr. Int..

[74] Shaban M., Elsayed Ramadan O.M., Zaky M.E., Mohamed Abdallah H.M., Mohammed H.H., Abdelgawad M.E. (2025). Enhancing Nursing Practices in Critical Care for Older Adults: A Systematic Review of Age-Friendly Nursing Interventions. J. Am. Med. Dir. Assoc..

[75] Alsabri M., Abady E., Elsnhory A.B., Aboali A.A., Rath S. (2026). Diagnostic accuracy of ultrasound for pediatric adnexal torsion: A systematic review and meta-analysis. Int. J. Emerg. Med..

[76] Tsai J., Lai J.Y., Lin Y.H., Tsai M.-H., Yeh P.-J., Chen C.-L., Chang Y.-J. (2022). Characteristics and risk factors for ischemic ovary torsion in children. Children.

[77] Oltmann S.C., Fischer A., Barber R., Huang R., Hicks B., Garcia N. (2009). Cannot exclude torsion—A 15-year review. J. Pediatr. Surg..

[78] Sancar S., Anayurt M., Dayı S. (2025). Surgical management of ovarian torsion in children: Detorsion, cyst/mass excision, and ovarian fixation. Reprod. Biol..

