# Supplementary material for: Ovarian Preservation Before Puberty: Surgical Management of Adnexal Torsion in Prepubertal and Prepubertal-Predominant Paediatric Cohorts—A Systematic Review and Meta-Analysis
Source: J Clin Med. 2026 Nov 6;15(21):8678. doi: 10.3390/jcm15218678 (PMC13764652; doi:10.3390/jcm15218678)
Supplement: Supplementary file 1 [file jcm-15-08678-s001.zip › Supplementary Files S1 and S4.pdf]

# Supplementary Material

***Ovarian Preservation Before Puberty: Surgical Management of Adnexal Torsion in Prepubertal and Prepubertal-Predominant Paediatric Cohorts—A Systematic Review and Meta-Analysis***

Hanadi Bakhsh

**Contents:** File S1 — database-specific search strategies. File S4 — completed PRISMA 2020 checklist. Files S2 and S3 (study-level extraction sheet, item-level critical appraisal, pooled estimates and the available screening/eligibility decision log) are supplied as a separate spreadsheet.

## File S1. Search strategies

Searches were executed on 2 September 2026. No language or date restrictions were applied. The strategy comprises three concept blocks combined with AND: (A) condition, (B) population, (C) surgical management.

### S1.1 Master strategy (as executed in Europe PMC)

Europe PMC indexes MEDLINE/PubMed, PubMed Central and preprint servers under a single query interface. Records retrieved: 1,985 (MEDLINE/PubMed), 77 (PubMed Central, non-MEDLINE), 13 (preprint servers).

```
("ovarian torsion" OR "adnexal torsion" OR "tubo-ovarian torsion" OR
 "torsion of the ovary" OR "ovarian torsions" OR "adnexal torsions")
AND
(prepubertal OR pre-pubertal OR prepubescent OR premenarchal OR premenarcheal
 OR child OR children OR childhood OR paediatric OR pediatric OR girl OR girls
 OR infant OR infants OR neonatal OR neonate)
AND
(surgery OR surgical OR laparoscopy OR laparoscopic OR laparotomy OR detorsion
 OR oophorectomy OR ovariectomy OR "salpingo-oophorectomy" OR adnexectomy
 OR oophoropexy OR cystectomy OR "ovarian preservation" OR "ovarian sparing"
 OR "ovary sparing" OR conservative OR salvage)
```

*Source-restricted counts were obtained by appending AND SRC:MED, AND SRC:PMC and AND SRC:PPR respectively.*

### S1.2 OpenAlex (multidisciplinary index)

Used to capture records not indexed in MEDLINE. Records retrieved: 737, of which 403 carried a PubMed identifier (and were removed as duplicates of the MEDLINE stream) and 334 did not.

```
filter=title_and_abstract.search:(ovarian torsion OR adnexal torsion)
  AND (prepubertal OR premenarchal OR children)
```

### S1.3 ClinicalTrials.gov (registry)

Records retrieved: 5 (2 observational, 3 interventional).

```
query.cond=ovarian torsion
query.term=children OR pediatric
```

### S1.4 Crossref (bibliographic verification)

Crossref was queried for every candidate citation to confirm authorship, journal, volume, pagination, year and digital object identifier before inclusion in the reference list. It was not used as a discovery source and contributed no records to the PRISMA flow.

## S1.5 Supplementary methods

Backward citation chasing: the reference lists of all included reports and of previous syntheses of paediatric ovarian torsion were screened. Forward citation chasing: reports citing the most frequently cited included cohorts were screened. Both were performed through Europe PMC and OpenAlex citation graphs.

## S1.6 Translations for databases not independently searched

The following translations are provided so that the review can be replicated or updated by investigators with subscription access. These databases were not independently searched in the present review; this is stated as a limitation in section 4.6 of the main manuscript.

**Embase (Ovid syntax)**

```
1  exp ovary torsion/
2  (ovarian adj2 torsion).ti,ab,kw.
3  (adnexal adj2 torsion).ti,ab,kw.
4  1 or 2 or 3
5  exp child/ or exp infant/ or exp school child/
6  (prepubert* or premenarch* or p?ediatric or child* or girl* or infant*).ti,ab,kw.
7  5 or 6
8  exp ovariectomy/ or exp laparoscopy/ or exp ovary surgery/
9  (detors* or oophorectom* or ovariectom* or oophoropex* or adnexectom* or
    cystectom* or "ovarian sparing" or "ovarian preservation" or salvage).ti,ab,kw.
10 8 or 9
11 4 and 7 and 10
```

**CINAHL (EBSCO syntax)**

```
(MH "Ovarian Diseases+") OR TI ("ovarian torsion" OR "adnexal torsion")
  OR AB ("ovarian torsion" OR "adnexal torsion")
AND (MH "Child+") OR (MH "Infant+") OR TI (prepubert* OR premenarch* OR child*
  OR girl* OR p#ediatric) OR AB (prepubert* OR premenarch* OR child* OR girl*)
AND TI (surg* OR laparoscop* OR detors* OR oophorectom* OR oophoropex*
  OR "ovarian sparing" OR "ovarian preservation") OR AB (same terms)
```

**Scopus (advanced search syntax)**

```
TITLE-ABS-KEY(("ovarian torsion" OR "adnexal torsion" OR "tubo-ovarian torsion"))
AND TITLE-ABS-KEY((prepubert* OR premenarch* OR child* OR p*ediatric OR girl*
  OR infant* OR neonat*))
AND TITLE-ABS-KEY((surg* OR laparoscop* OR laparotom* OR detors* OR oophorectom*
  OR ovariectom* OR adnexectom* OR oophoropex* OR cystectom* OR "ovarian sparing"
  OR "ovarian preservation" OR conservat* OR salvage))
```

**Web of Science Core Collection**

```
TS=("ovarian torsion" OR "adnexal torsion" OR "tubo-ovarian torsion")
AND TS=(prepubert* OR premenarch* OR child* OR pediatric OR paediatric OR girl*
  OR infant* OR neonat*)
AND TS=(surg* OR laparoscop* OR detors* OR oophorectom* OR ovariectom*
  OR adnexectom* OR oophoropex* OR cystectom* OR "ovarian sparing"
  OR "ovarian preservation" OR conservat* OR salvage)
```

**Cochrane CENTRAL**

```
#1 MeSH descriptor: [Ovarian Diseases] explode all trees
#2 ("ovarian torsion" OR "adnexal torsion"):ti,ab,kw
```

```
#3 #1 OR #2
#4 (prepubert* OR premenarch* OR child* OR pediatric OR paediatric OR girl*):ti,ab,kw
#5 (surg* OR laparoscop* OR detors* OR oophorectom* OR oophoropex*):ti,ab,kw
#6 #3 AND #4 AND #5
```

## File S4. PRISMA 2020 checklist

*Completed against Page MJ, McKenzie JE, Bossuyt PM, et al. The PRISMA 2020 statement: an updated guideline for reporting systematic reviews. BMJ. 2021;372:n71. Section numbers refer to the main manuscript.*

| Item | Topic | Checklist item | Reported in |
|---|---|---|---|
| 1 | Title | Identify the report as a systematic review. | Title page |
| 2 | Abstract | See the PRISMA 2020 for Abstracts checklist. | Abstract |
| 3 | Rationale | Describe the rationale for the review in the context of existing knowledge. | 1. Introduction, paras 1–5 |
| 4 | Objectives | Provide an explicit statement of the objective(s) or question(s). | 1. Introduction, RQ1–RQ4 |
| 5 | Eligibility criteria | Specify the inclusion and exclusion criteria and how studies were grouped. | 2.2 |
| 6 | Information sources | Specify all databases, registers and other sources searched, and the date each was last searched. | 2.3 |
| 7 | Search strategy | Present the full search strategies for all sources. | 2.3; Supplementary S1 |
| 8 | Selection process | Specify the methods used to decide whether a study met the inclusion criteria. | 2.4; Supplementary S3 |
| 9 | Data collection process | Specify the methods used to collect data from reports. | 2.5; Supplementary S2 |
| 10a | Data items — outcomes | List and define all outcomes for which data were sought. | 2.2 (Outcomes) |
| 10b | Data items — other variables | List and define all other variables for which data were sought. | 2.5 |
| 11 | Study risk of bias assessment | Specify the methods used to assess risk of bias. | 2.6; Figure 6; Supplementary S2g |
| 12 | Effect measures | Specify for each outcome the effect measure used in the synthesis or presentation of results. | 2.2; 2.7 |
| 13a | Synthesis — eligibility | Describe the processes used to decide which studies were eligible for each synthesis. | 2.2; 3.1 |
| 13b | Synthesis — data preparation | Describe any methods required to prepare the data for presentation or synthesis. | 2.5; 2.7 |
| 13c | Synthesis — tabulation/visual | Describe any methods used to tabulate or visually display results. | 2.7; Tables 1–6; Figures 1–7 |
| 13d | Synthesis — methods | Describe any methods used to synthesise results and provide a rationale for the choices. | 2.7 |
| 13e | Synthesis — heterogeneity | Describe any methods used to explore possible causes of heterogeneity. | 2.7 (subgroups, meta-regression) |
| 13f | Synthesis — sensitivity | Describe any sensitivity analyses conducted. | 2.7; 3.3; 3.7 |
| 14 | Reporting bias assessment | Describe any methods used to assess risk of bias due to missing results. | 2.7; 3.7; Figure 4 |
| 15 | Certainty assessment | Describe any methods used to assess certainty in the body of evidence. | 2.9; Table 4 |
| 16a | Study selection — flow | Describe the results of the search and selection process. | 3.1; Figure 1 |
| 16b | Study selection — exclusions | Cite studies that met many but not all inclusion criteria and explain why they were excluded. | Supplementary S3 |
| 17 | Study characteristics | Cite each included study and present its characteristics. | 3.2; Table 1 |
| 18 | Risk of bias in studies | Present assessments of risk of bias for each included study. | 3.7; Figure 6; Supplementary S2g |
| 19 | Results of individual studies | Present, for all outcomes, summary statistics for each study. | Figures 2 and 3; Supplementary S2a–S2d |
| 20a | Results of syntheses — summary | Summarise the characteristics and risk of bias among contributing studies. | 3.2; 3.7 |
| 20b | Results of syntheses — estimates | Present results of all statistical syntheses with summary estimates, precision and heterogeneity. | 3.3–3.6; Tables 2 and 3 |

| Item | Topic | Checklist item | Reported in |
|---|---|---|---|
| 20c | Results of syntheses — heterogeneity | Present results of all investigations of possible causes of heterogeneity. | 3.3; Figure 5; Table 2 |
| 20d | Results of syntheses — sensitivity | Present results of all sensitivity analyses. | 3.3; 3.7 |
| 21 | Reporting biases | Present assessments of risk of bias due to missing results. | 3.7; Figure 4 |
| 22 | Certainty of evidence | Present assessments of certainty in the body of evidence for each outcome. | 3.10; Table 4 |
| 23a | Discussion — interpretation | Provide a general interpretation of the results in the context of other evidence. | 4.1; 4.2 |
| 23b | Discussion — limitations of evidence | Discuss any limitations of the evidence included. | 4.6 |
| 23c | Discussion — limitations of review | Discuss any limitations of the review processes used. | 4.6 |
| 23d | Discussion — implications | Discuss implications for practice, policy and future research. | 4.4; 4.7 |
| 24a | Registration | Provide registration information, including the register name and registration number. | 2.1; Declarations |
| 24b | Protocol | Indicate where the protocol can be accessed, or state that one was not prepared. | 2.1 |
| 24c | Amendments | Describe and explain any amendments to the information provided at registration. | Section 2.1 (post-registration amendments listed and explained) |
| 25 | Support | Describe sources of financial or non-financial support and the role of funders. | Declarations (Funding) |
| 26 | Competing interests | Declare any competing interests of review authors. | Declarations |
| 27 | Availability of data, code | Report which of the following are publicly available and where. | Declarations |
